# Multiple mitogenomes indicate Things Fall Apart with Out of Africa or Asia hypotheses for the phylogeographic evolution of Honey Bees (*Apis mellifera*)

**DOI:** 10.1038/s41598-023-35937-4

**Published:** 2023-06-09

**Authors:** Steven M. Carr

**Affiliations:** Genetics, Evolution, and Molecular Systematics Laboratory, Department of Biology, Memorial University of Newfoundland, St John’s, NL A1C 5S9 Canada

**Keywords:** Evolutionary genetics, Phylogenetics

## Abstract

Previous morpho-molecular studies of evolutionary relationships within the economically important genus of honey bees (*Apis*), including the Western Honey Bee (*A. mellifera* L.), have suggested Out of Africa or Asia origins and subsequent spread to Europe. I test these hypotheses by a meta-analysis of complete mitochondrial DNA coding regions (11.0 kbp) from 22 nominal subspecies represented by 78 individual sequences in *A. mellifera*. Parsimony, distance, and likelihood analyses identify six nested clades: Things Fall Apart with Out of Africa or Asia hypotheses. Molecular clock-calibrated phylogeographic analysis shows instead a basal origin of *A. m. mellifera* in Europe ~ 780 Kya, and expansion to Southeast Europe and Asia Minor ~ 720 Kya. Eurasian bees spread southward via a Levantine/Nilotic/Arabian corridor into Africa ~ 540 Kya. An African clade re-established in Iberia ~ 100 Kya spread thereafter to westerly Mediterranean islands and back into North Africa. Nominal subspecies within the Asia Minor and Mediterranean clades are less differentiated than are individuals within other subspecies. Names matter: paraphyletic anomalies are artefacts of mis-referral in GenBank of sequences to the wrong subspecies, or use of faulty sequences, which are clarified by inclusion of multiple sequences from available subspecies.

## Introduction

The genus *Apis* comprises at least a dozen nominal species of Honey Bees found throughout Asia, Africa, the Middle East, and Europe, including the type species *A. mellifera* L., the Western Honey Bee (Table 1)^1,2^. Although several species are important as traditional local sources of honey, only two cavity-nesting species, *A. cerana* in India and *A. mellifera*, have historically been domesticated for this purpose. Apiculture of *A. mellifera* is documented in Egypt as far back as 2600 BCE^3^. (References to the Levant as “*a land of milk and honey*” (Exodus 3:8) may refer instead to syrup from wind- and (or) hand-pollinated oasis dates (*Phoenix dactylifera*) of even greater antiquity^3^). *A. mellifera* has been transported by humans around the world as the principal commercial source of honey and beeswax, and is also of particular agricultural importance in the Americas as an introduced pollinator of certain vegetable and fruit crops that are themselves introduced^4^. Competition between introduced generalist honey bees and specialist native insect pollinator (including other non-apine bees) for pollen (“*pollen theft*”) may be detrimental to native plant species^5^. Geographic variation among different local strains or subspecies of *A. mellifera* is known to contribute to this impact, as it does quality and quantity of honey and other behaviors^6^. Notably, so-called “*Africanized*” bees have resulted from the escape of African subspecies crossed into domesticated South American hives, where the hybrids combine increased yield of honey with greater aggressiveness, and pose a hazard to humans^7^.

**Table 1:** *Apis* taxonomy, GenBank accessions, & distribution of species and subspecies for which complete mitogenome sequences are available^2^. The subspecies epithet *iberica* is a junior synonym for the Caucasian form, and as such is pre-empted for the Iberian Honey Bee. The correct trinomial is *A. m. iberiensis* Engel, 1999. The subspecies epithet for the Caucasian Honey Bee is given variously as *caucasica* or *caucasia* or: the latter is correct.

Subgenus	Species and subspecies	Authority	Common name	GenBank	Range and comments
*Micrapis*			Dwarf honey bees		
	*A. florea*	Fabricius, 1787	Red dwarf honey bee	JK982136	Widespread in S Asia, across India to Middle East & Africa
	*A. andreniformis*	Smith, 1858	Black dwarf honey bee	KF361157	SE Asia
*Megapis*			Giant honey bees		
	*A. dorsata*	Fabricius, 1793	Giant Honey Bees	KX908207	
	*A. laboriosa*	(Smith, 1871)	Himalayan honey bee	AP018039	Himalayas; former subspecies of *A. dorsata*
*Apis*			Cavity-nesting honey bees		
	*A. koschevnikovi*	(Enderlein, 1906)	Koschevnikovi honey bee	KY348372	Former subspecies of *A. cerana*; restricted to Borneo
	*A. nuluensis*	(Tingek, Koeniger, & Koeniger, 1996)	Bornean honey bee	AP018157	Former subspecies of *A. cerana*
	*A. cerana*	Fabricius, 1793	Eastern honey bee	KX908206	Domesticated in SE & E Asia
	*A. nigrocincta*	Smith, 1861	Philippine honey bee	AP018370	Confined to Phillipines
	*A. mellifera*	Linnaeus, 1761	Western honey bee		
	*A. m. mellifera*	Linnaeus, 1758	German honey bee	KX926884 et al	N Europe; domesticated worldwide
	*A. m. sinisxinyuan*	Chen et al., 2016	Xinyuan honey bee	MN733955	Xinjiang province, China
	*A. m. ligustica*	Spinola, 1806	Italian honey bee	NC001566 et al	Italy; domesticated worldwide
	*A. m. carnica*	Pollmann, 1879	Carniolan honey bee	MN250878	Slovenia, E Alps, & N Balkans
	*A. m. carpatica*	Pollmann, 1879	Carpathian honey bee	AP018403	Carpathia
	*A. m. meda*	Skorikov, 1929	Persian honey bee	KY464958	Iran
	*A. m. caucasia*	Pollmann, 1879	Cacucasian honey bee	AP018044	Cent Caucasus
	*A. m. anatolica*	Maa, 1953	Anatolian honey bee	MT188686	Anatolia in Turkey
	*A. m. syriaca*	Skorikov, 1929	Syrian honey bee	KP163643	Near East & Israel
	*A. m. lamarckii*	Cockerell, 1906	Egyptian honey bee	KY464957	Nile Valley
	*A. m. jemenitica*	Ruttner, 1996	Arabian honey bee	MN714161	Somalia, Uganda, Sudan, Yemen
	*A. m. simensis*	Meixner et al. 2011	Ethiopian honey bee	MN585108	Ethiopia
	*A. m. unicolor*	Latreille, 1804	Madagascaran honey bee	MN119925	Confined to Madagascar
	*A. m. ibieriensis*	Engel, 1999	Spanish honey bee	MN585110	Iberian Peninsula
	*A. m. ruttneri*	Sheppard, Arias, Grech, & Meixner, 1997	Maltese honey bee	MN714162	Confined to Maltese Islands
	*A. m. intermissa*	von Buttel-Relepen, 1906	Tunisian honey bee	KM458618	N Africa
	*A. m. sahariensis*	Baldensperger, 1932	Saharan honey bee	MF351881	Moroccan desert oases
	*A. m. scutellata*	Lepeltier, 1836	East African lowland honey bee	KJ601784 et al	Cent & E Africa; source of Africanized *A. m. mellifera*
	*A. m. capensis*	Eschscholtz, 1822	Cape honey bee	KX870183 et al	Cape region of South Africa
	*A. m. monticola*	Smith, 1961	East African mountain honey bee	MF678581	Mountains of E Africa
	*A. m. adansonii*	Latreille, 1804	West African honey bee	MN585109	Nigeria & Burkina Faso

Morpho-behavioral taxonomy has recognized three species groups within the genus: Dwarf Honey Bees (*A. florea* and *A. andreniformis*), Giant Honey Bees (*A. laboriosa*, *A. dorsata*, and *A. breviligula*), and Cavity-Nesting Honey Bees (*A. mellifera* together with *A. nuluensis*, *A. nigrocincta*, *A. cerana* (including *A. indica*), and *A. koschevnikovi*). *A. mellifera* is the only species with a native range in Africa and Europe, and includes more than 30 nominal subspecies^2^. These have historically been clustered as four continental groups, designated **ACMO** for African, Continental, Mellifera, and Oriental distributions^8^. The geographic origin and evolutionary spread of these groups remains controversial, as has their resolution into ancestor–descendant phylogenetic lineages. Molecular studies based on analyses of various components of the nuclear genome (see Discussion) agree broadly on rearrangement of these as an **MAOC** backbone, rooted so as to offer alternative theories of origin, either “*Thrice Out of Africa*”^9^ [root within **A**] or “*Thrice Out of Asia*”^10^ [root between **A** & **O**]. These studies are based on fewer than a dozen of the available subspecies of *A. mellifera*. Other phylogeographic conclusions have been reached from analyses of complete mitochondrial DNA (mtDNA) genomes from single sequences per subspecies^11,12^. In particular, Tihelka and co-workers in this journal^12^ have recently provided a meta-analysis of mtDNA, based on a substantial body of data from Boardman et al.^11^ and new methods of phylogenetic inference. They reached yet another alternative hypothesis, a Middle Eastern/North African origin of *A. mellifera*.

Tihelka and co-workers emphasize the need for a reliable backbone phylogeny for *A. mellifera* L. to understand the evolution of the subspecies, including its geographic origin and the development of adaptative differences among subspecies that contribute to their ecological and commercial success^6^. My editorial review of the use of an mtDNA spacer region between the CO1 and CO2 genes as a tool for discrimination of individual bees within and among subspecies of *A. mellifera* [cf. Ref.^13^] found no comprehensive phylogenetic evaluation that included multiple mtDNA genome variants within multiple subspecies^11,12,14–26^. Unexpectedly, my preliminary assembly of multiple sequences assigned to *A. m. mellifera* in GenBank, taken at face value, seemed to indicate extensive para- and (or) polyphyly and (or) extraordinary genetic diversity within this subspecies and with respect to other taxa.

In the twenty-first century CE and the third century L., the importance of classical “*alpha*” taxonomy^27^ in finding, describing, and naming taxa remains critical. I provide here a phylogeographic re-evaluation of the evolution of *Apis*, based on a meta-analysis of the 13 coding regions (11,006 bp) from available mitogenomes in nine species of the genus *Apis*, including 78 individuals from 22 subspecies of the type species *A. mellifera* L. This meta-analysis indicates that Things Fall Apart with Out of Africa, Asia, and Middle East hypotheses, in favor of a European origin.

## Materials and methods

### Sequence data

Complete mitochondrial DNA genomes of taxa within the genus *Apis* were identified in the GenBank Taxonomic library by selection of “Genome”-flagged sequences within the display of species and subspecies. This was supplemented by a search of the “Nucleotide” library with the search term “Apis mitochondrion” through the end of October 2022. Table 1 lists GenBank accession numbers for sequences from nine species of *Apis* and 22 subspecies of *A. m. mellifera* as used here, together with the subspecies’ geographic provenance.

The mitogenomic sequence of a curated Norwegian specimen of *A. m. mellifera* (KY926884) was used as the alignment reference. Alignment was done by eye with the help the MEGA X program^28^. The *Apis* mitochondrial coding genome comprises 13 genes over 11,043 bps (Table 2). The GenBank annotations of various authors delimit coding regions with slightly different 5′ and 3′ endpoints, especially among species: spaces were inserted between coding regions so as to preserve open reading frames. The light-strand coding regions of the ND5, ND4, ND4L, and ND1 genes were included in their sense-strand equivalent 5′ → 3′ coding directions.

**Table 2:** *Apis* species & subspecies mitochondrial genome organization. Coding Regions for comparisons among species [left] are listed in their 5′ → 3′ coding order, with light-strand coding regions (italics) as their reverse complements. Coding regions for comparisons of subspecies of *A. mellifera* with reference to *A. m. mellifera* [right] are shown in their 5′ → 3′ order on the heavy strand, with light strand regions described from the heavy strand. Differences between species and subspecies gene region and triplet lengths are due to indels necessary to maintain spacing.

Order	Gene	*Apis* species coding regions			*A. mellifera* subspecies, 5'—3' heavy strand order			
		N-term	Bases	Triplets	5′ sequence	5′ end	Bases	Triplets
1	ND2	IFFMN	1002	334	ATCTTCTTCAT	IFFMN	1002	334
2	CO1	MMKWF	1563	521	ATAATAAAGTG	MMKWF	1560	520
3	CO2	ISTWF	678	226	ATTTCCACATG	ISTWF	678	226
4	ATP8	IPQMM	162	54	ATTCCTCAAA	IPQMM	138	46
5	ATP6	MKLTL	681	227	ATGAAATTGAC	MKLTL	681	227
6	CO3	MKKNF	786	262	ATGAAAAAAAAT	MKKNF	780	260
7	ND3	MSFIF	354	118	ATAAGATTT	MSFIF	354	118
8	*ND5*	IIKMM	1668	556	*TTAAAAATTCAT*	LKIH*	1665	555
9	*ND4*	MLMMS	1314	438	*TTAAATAAAATA*	*LNKMK*	1347	449
10	*ND4L*	IKLLF	264	88	*TTAATAAATCAA*	*LMNQI*	264	88
11	ND6	IMLTI	522	174	ATCATATTAAC	IMLTI	504	168
12	CYTB	MKKFM	1152	384	ATGAAAAAATTT	MKKFM	1152	384
13	*ND1*	IWVLI	924	308	*TTAAATACATAA*	LNT*K	918	306
	Totals		11,070	3690			11,043	3681

The 3′ region of the ND4 heavy-strand equivalent is difficult to align across subspecies, and a 37 bp region was excluded from all analyses. Ambiguous base calls (mostly W for A/T, flanked by AT bases) in several sequences have been tacitly resolved, such that these positions are invariant over all sequences. Several autapomorphic insertions of single triplets among species and subspecies have been tacitly removed. The coding region mitogenome sequence for a Bumble Bee, *Bombus ignitus* (GenBank NC010967), was used as the outgroup for inter-species comparisons. Honey bees and Bumble Bees occur in the tribes Apini and Bombini, respectively, of the subfamily Apinae. The alignment of coding regions between *Bombus* and *Apis* spp. is in some areas speculative: experimental inclusion or exclusion of ambiguous regions does not affect the inferred branching order of *Apis* species, and does not materially affect its statistical support. The consensus alignment for analysis comprises 11,006 bases within *A. mellifera* subspecies, and 11,070 bases across *Apis* and *Bombus*.

### Phylogenomic analysis

I performed three forms of phylogenetic analyses with MEGA X^28^. Maximum Parsimony (MP) analyses was performed with all nucleotide positions equally weighted, and SPR search. Maximum Parsimony analysis is foregrounded to identify inter-subspecific SNPs and to provide intra-specific patristic differences within *Apis*, and intra- *versus* inter-subspecific differences within *A. mellifera*. Maximum Likelihood analyses (ML) was performed with the general time reversible (GTR) model allowing for invariant sites and nearest-neighbor interchange (NNI) search. For molecular clock calculations, I used a fixed rate of 0.0115 substitutions/site/Myr on node distances calculated in a linearized Maximum Likelihood tree RelTime-ML model in MEGA (see “Discussion”). Neighbor Joining (NJ) analysis was performed on counts of differences, with the maximum composite likelihood model, and SPR search. Statistical confidence in all three methods was estimated from 3000 bootstrap replications each, under the same conditions as the main search. Concern has been expressed^12^ as to the effect of inclusion of third triplet-position bases on phylogenetic inference. I examined the effect of exclusion of these data (model P12 and modifications thereof^12^) in all three analyses.

Initial assessment of the available mitogenomes for *A. mellifera* identified 16 Arabian bees in four monophyletic clades assigned to *A. m. mellifera* GenBank accessions MT745901–MT745915^14^ that comprise sets of 5, 4, 4, and 3 identical sequences each: one sequence was included from each set, and the rest tacitly. A set of 11 mitogenomes from Kenyan bees (KJ396181–KJ396191)^15^ assigned in GenBank to *A. m. mellifera* are used here with their correct subspecies identifications as provided by Z. Fuller (pers. comm.). A series of 20 sequences attributed to *A. m. capensis* (MG552683–692) or *A. m. scutellata* (MG552693–702) was retained in full. GenBank accessions KY926882 and KY926883 are attributed to *A. m. syriaca* and *A. m. intermissa*, respectively. Inspection of both sequences indicates numerous anomalies in coding region that produce branch attraction between the two and large phylogenetic separations from other GenBank accessions assigned to the same subspecies (Supplementary Fig. S1). Both are excluded from primary analyses here.

All figures were drawn with Corel PaintShop Pro 2023 (version 25).

## Results

**Figure 1:**
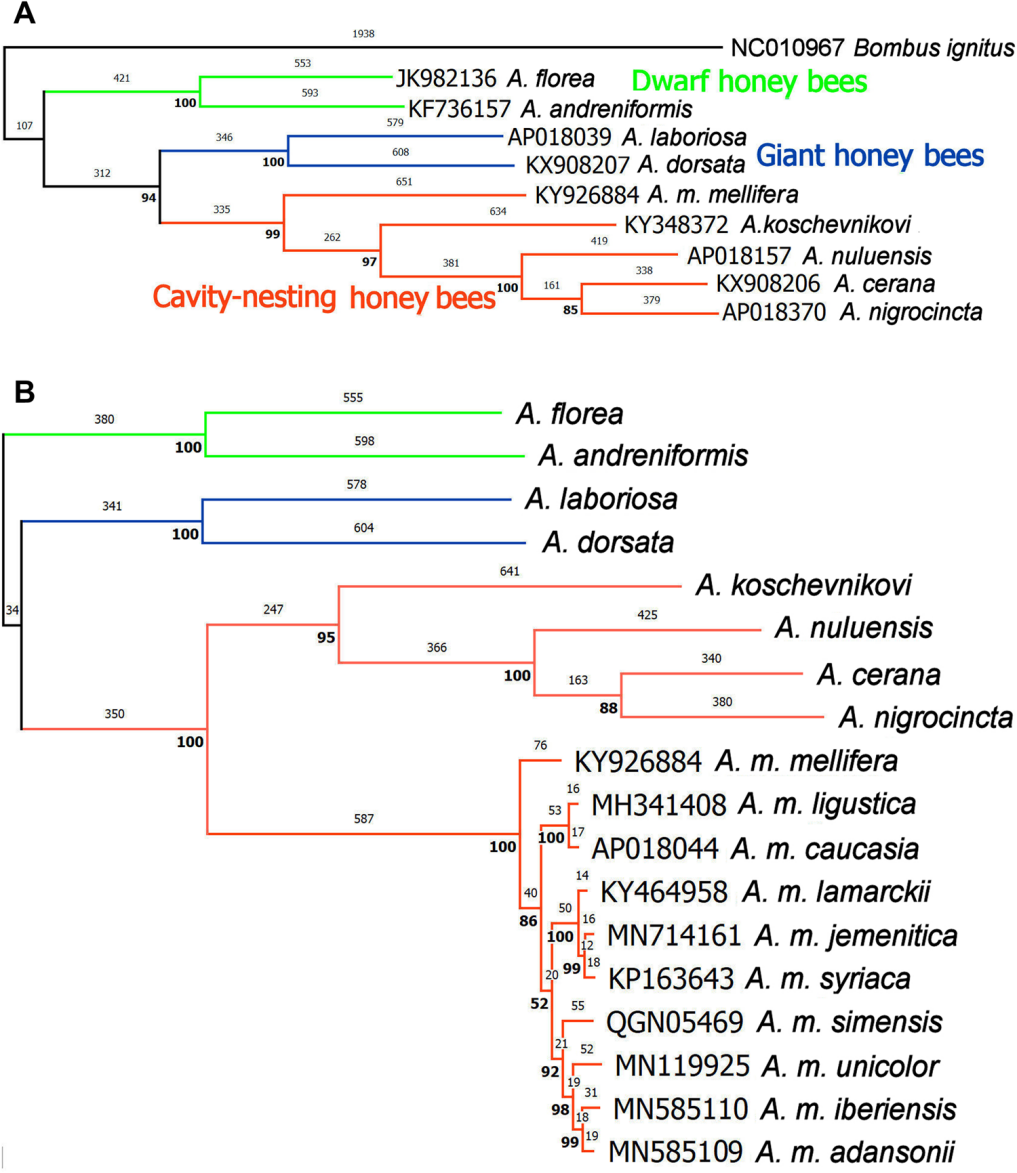
Maximum Parsimony analysis of phylogenetic relationships among mtDNA genomes sequences of nine species of *Apis* honey bees. (**a**) The tree is rooted with a bumblebee *Bombus ignitus* as outgroup. *A. m. mellifera* (KY926884) is the basal-most member of that species and the alignment reference. Numbers above branches are inferred numbers of nucleotide substitutions; numbers in bold below branches are percent support in 3000 bootstrap replicates. Identical branching order and substantially similar bootstrap support are given by Maximum Likelihood and Neighbor Joining methods. (**b**) As above, with removal of *Bombus* and addition of key subspecies of *A. mellifera* (cf. Fig. 2).

Figure 1a shows the MP analysis for nine species of *Apis* (Apini) with *Bombus ignitus* (Bombini) as outgroup. Out of seven interspecies nodes, six are supported by > 94% of bootstraps. Analyses with the NJ and ML methods give identical branching orders and substantially similar bootstrap support. Within *Apis*, the Dwarf Honey Bee pair [*A. florea* + *A. andreniformis*] is the outgroup to the remaining taxa. Within the Cavity-Nesting species, *A. mellifera* is the sister species to the others, and its phenetic difference from other cavity-nesting species is greater than that between the Dwarf and Giant Honey Bee pairs. Figure 1b shows the MP analysis of *Apis* species alone including ten subspecies of *Apis mellifera*: *A. m. mellifera* is sister to the remaining subspecies. Note that *A. florea* and *A. mellifera*, the only species whose native ranges overlap in the Middle East, are in separate morpho-behavioral and molecular clades.

**Figure 2:**
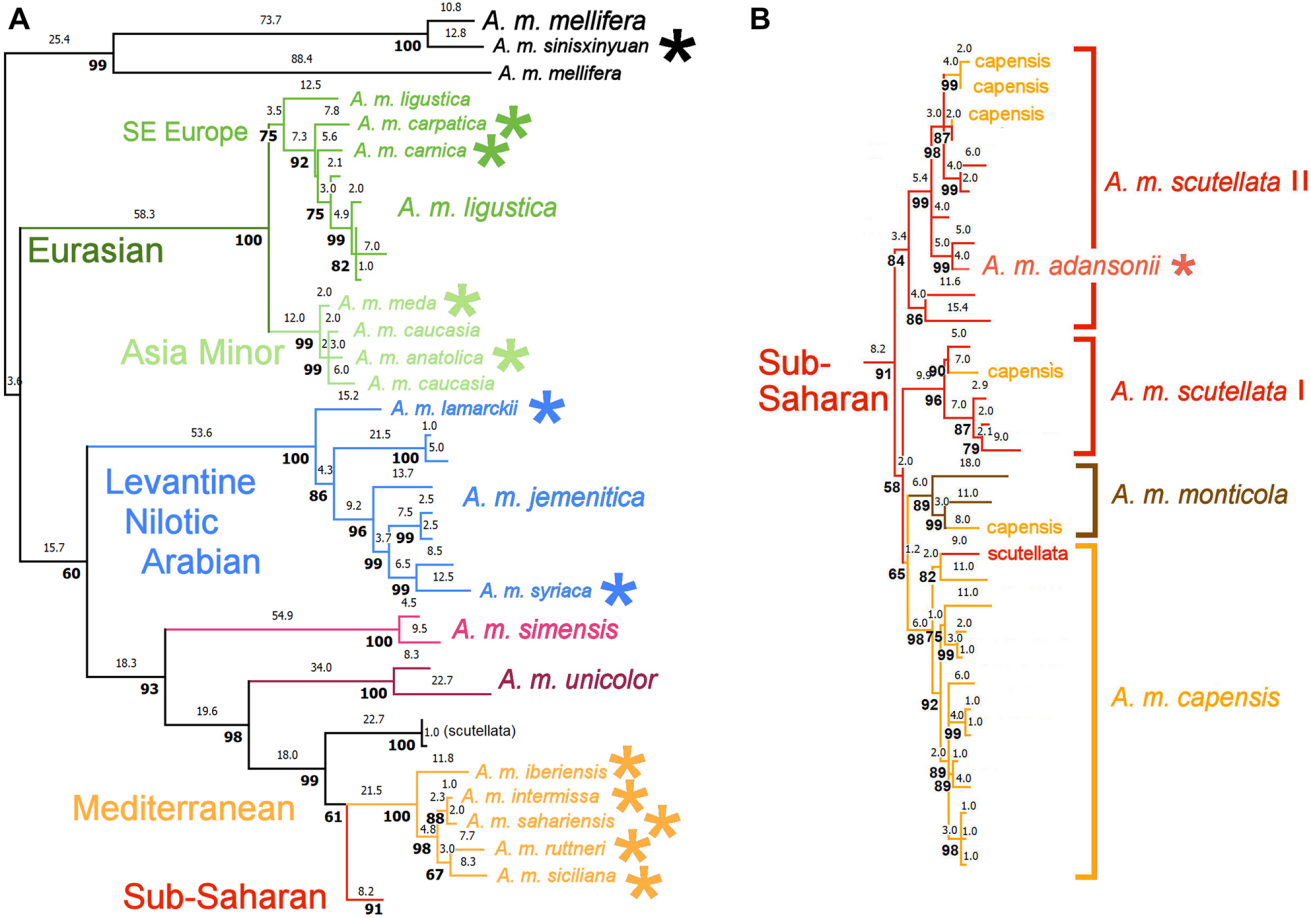
Schematic maximum parsimony analysis of phylogenetic relationships among mtDNA genome sequences of 66 individual *A. mellifera* honey bees from 22 subspecies. Rooting as indicated by Fig. 1. Numbers above branches are inferred numbers of nucleotide substitutions; numbers in bold below branches are percent support in 3000 bootstrap replicates, with SPR branching swapping. The tree shown is one of nine minimum length trees that differ only by rearrangements at unresolved nodes. Sequences in the Sub-Saharan clade that make that subspecies paraphyletic are tagged in Roman font, as are two sequences referred to *A. m. scutellata* that are outside that clade. An additional 12 sequences from the Arabian series that are identical to the four shown are not included. Sequences curated as *A. m. mellifera* in GenBank^15^ are re-assigned their proper names in parentheses (J. M. Fuller, pers. comm). Subspecies represented by single sequences are indicated by (*). Named phylogeographic clades discussed in the text are indicated in color. The complete MP tree with GenBank accession numbers is given in Supplementary Fig. S1, along with those for Maximum Likelihood and Neighbor Joining methods (Supplementary Figs. S2 and S3, respectively).

Figure 2 shows the schematic MP analysis of 66 (+ 12 *tacit*) sequences from 22 subspecies of *A. mellifera*, with multiple sequences included as described in Methods. The tree is rooted with *A. m. mellifera* as obtained from the analysis shown in Figs. 1b. MP, ML and NJ analyses (Supplementary Figs. S1–S3, with complete GenBank accession numbers) give the same branching order among subspecies and most multiple individuals within subspecies, and substantially similar bootstrap support.

**Figure 3:**
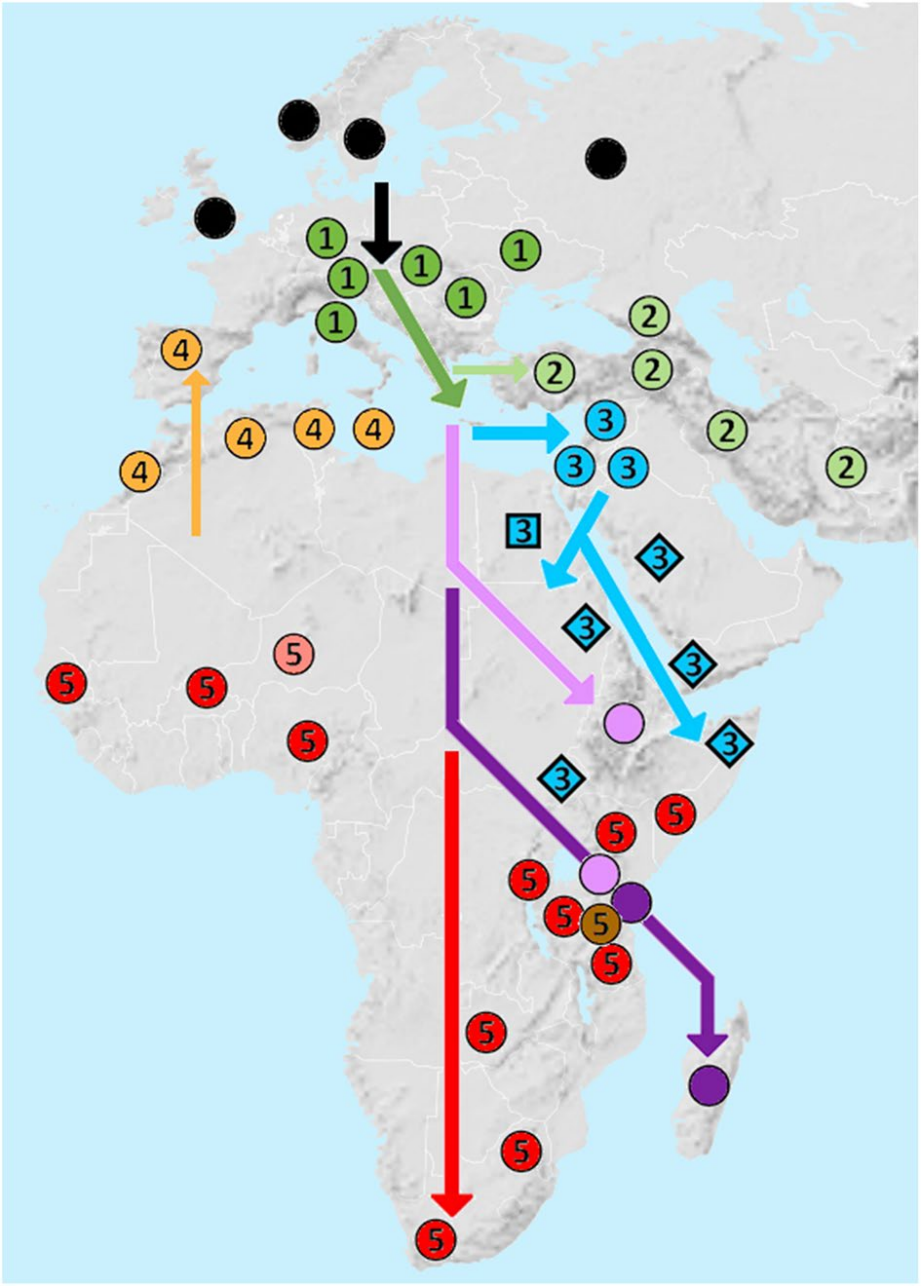
Phylogeographic evolution in context of geographic distribution^2^ of subspecies of *A. mellifera* as inferred from mitogenomic data. Numbered symbols indicate five clades described in the text and in Fig. 2. Dark and light green circles indicate respectively subspecies in the  Southeast European and  Asia Minor clades included within the Eurasian superclade. Blue symbols  indicate the Levantine (circles), Nilotic (squares), and Arabian (*A. m. jemenitica*) (diamonds) clades. Light and dark purple circles indicate independent *A. m. simensis* and *A. m. unicolor* lineages, respectively. Light orange symbols  indicate subspecies in the Mediterranean clade. Red circles  indicate the paraphyletic assemblage of *A. m. scutellata* and *A. m. capensis*, including *A. m. adansonii* (light red) and *A. m. monticola* (brown). Base map modified from [https://commons.wikimedia.org/wiki/File:BlankMap-World.svg].

The consensus branching order identifies a series of well-defined, successively nested phylogeographic clades, with European *A. m. mellifera* sensu lato ([[KY926884 + *A. m. sinisxinyuan*] + Arabian MT745913]; MT745912, 914, and 915 not shown) basal to the remainder. Their geographic provenances are as follows (Fig. 3):

I. Southeast European = [*A. m. ligustica* + [*A. m. carnica* + *A. m. carpatica*]]. The pairwise patristic difference between *A. m. carnica* + *A. m. carpatica* (14) is less than the maximum between variant *A. m. ligustica* sequences (32).
II. Asia Minor = [*A. m. meda* + [*A. m. caucasia* + [*A. m. anatoliaca*]]], with both nodes supported > 95%. Maximum pairwise patristic differences between subspecies sequences (5–10) are also less than those within *A. m. ligustica*.
III. Levantine/Nilotic/Arabian = [*A. m. lamarckii* + [*A. m. jemenitica* + [*A. m. syriaca*]]] as three separate subspecies lineages. The pairwise patristic difference between *A.m. lamarckii* and *A. m. syriaca* (44) is less than the maximum within *A. m. jemenitica* (49).
IV. Mediterranean = [*A. m. iberiensis* + [[*A. m. intermissa* + *A. m. sahariensis*] + [*A. m. ruttneri* + *A. m. siciliana*]]]], with all nodes supported > 99%. The mainland European variant is basal to the North African variants, and the two island variants are paired. Maximum pairwise differences between subspecies sequences (3 ~ 25) are less than those within *A. m. ligustica* or *A. m. jemenitica*.
V. Sub-Saharan = paraphyletic assemblage of sequences assigned to *A. m. scutellata* and *A. m. capensis*. *A. m. scutellata* occurs in two clades, one of which is more closely related to *A. m. capensis*. Monophyly of either subspecies is further confounded by scattered referral of individual sequences to the other subspecies across these three clades. The single *A. m. adansonii* type is paired with an *A. m. scutellata* sequence. The two *A. m. monticola* variants encompass a third assigned to *A. m. capensis*.

The (SE European + Asia Minor) clades constitute an inclusive Eurasian superclade, and an African clade can be recognized as [Ethiopian *A. m. simensis* + [Malagasy *A. m. unicolor* + [Mediterranean + Sub-Saharan]]], allowing for the mainland European and insular taxa in the Mediterranean clade.

Pairwise differences between all nominal subspecies represented by single sequences within Clades I, II, and IV are smaller than those between replicate sequences in the same or sister lineages (cf. *A. m. ligustica*, *A. m. jemenitica*, *A. m. scutellata*, and *A. m. capensis*). The same is true for a single sequence from *A. m. adansonii* (from Niger) with respect to *A. m. scutellata*, and two sequences from *A. m. monticola* (from the east African mountains) with respect to *A. m. capensis* in the Sub-Saharan lineage.

Small but consistent majorities of bootstraps in all phylogenetic methods supports the Levantine/Nilotic/Arabian clade as sister to the African clade, and the Mediterranean clade as sister to the Sub-Saharan clade. A pair of near-identical Kenyan bee sequences (KJ396184 and KJ396190)^15^ referred to *A. m. scutellata* by Z. Fuller (pers. comm.) is cladisitically separate from other *scutellata* and both the Mediterranean and Sub-Saharan clades; its exact position varies slightly among methods.

**Figure 4:**
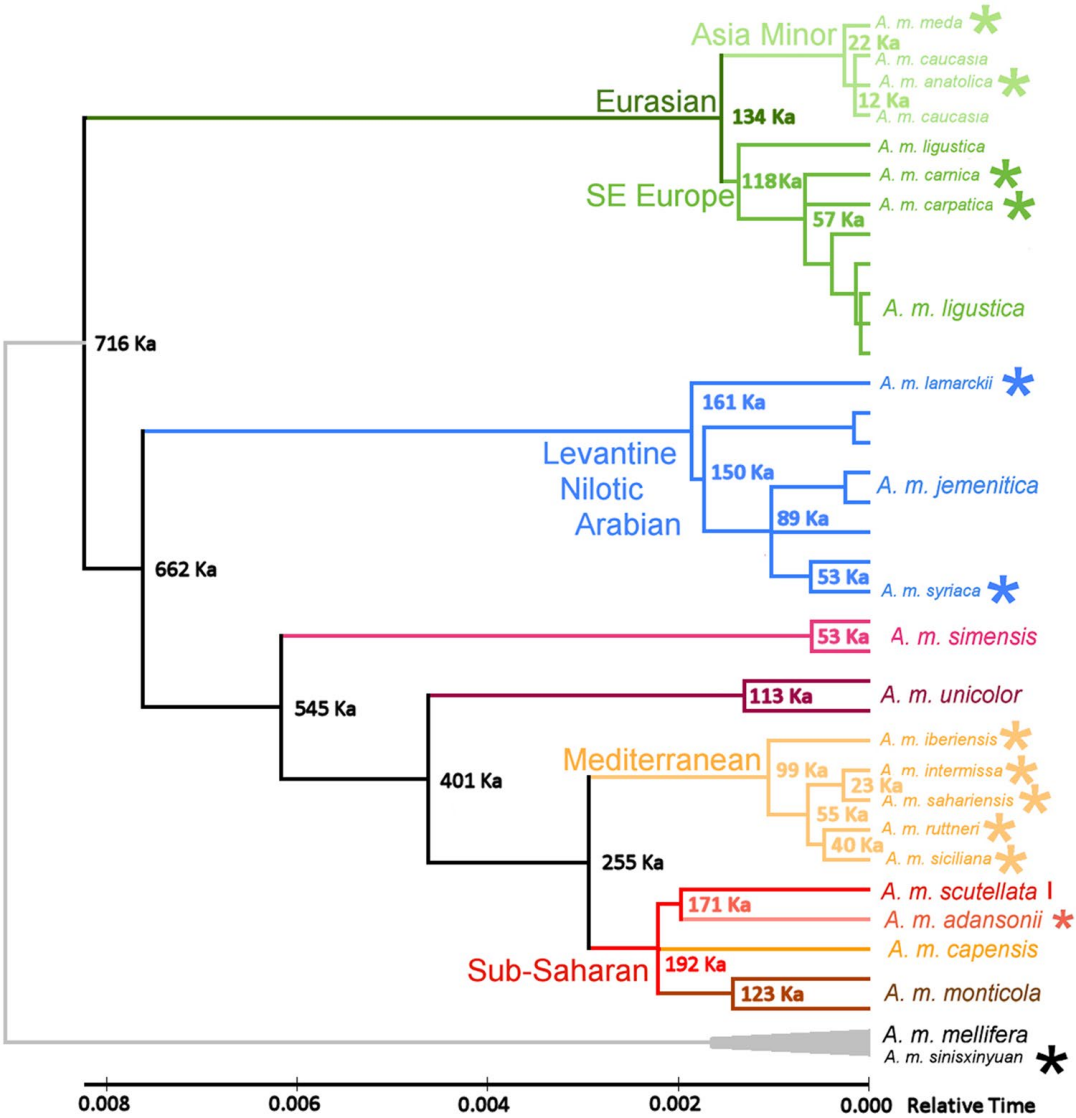
An mtDNA-based molecular clock for within- and among-subspecies divergences of *A. mellifera*. Sequences are coded as in Fig. 2: only one representative each of *A. m. scutellata* and *A. m. capensis* is included (n = 37). Divergence times are calculated from a linearized ML model with *A. m. mellifera* sequences as the designated outgroup (cf. Supplementary Fig. S2). The clock is calibrated from the mean linearized nucleotide subs/site distances to each node (Relative Time) at 0.0115 subs/site/Myr (see text for sample calculation). See Supplementary Fig. S4 for the clock of *A. mellifera* within *Apis*, with *Bombus* as the designated outgroup.

With a fixed clock rate of 0.0115 subs/site/Myr (see “Discussion”), all node distances in linearized ML trees can be converted directly to their age of divergence in a molecular clock (Fig. 4). For example, given a calculated distance to the basal node in Fig. 4 of 0.008234 subs/site (Relative Time), the node is dated at (0.008234 subs/site)/(0.0000115 subs/site/Kyr) = 716 Ka. For *Bombus* and other *Apis* species included as in Fig. 1, the molecular clock indicates a late Miocene radiation (6–11 Mya) of morpho-behavioral groups within *Apis* (Supplementary Fig. S4), and a European origin of *A. mellifera* 780 Kya in the late Pleistocene. The molecular clock for subspecies of *A. mellifera* is shown in Fig. 4 for a subset of 37 sequences included in Fig. 2a, with single representative sequences for *A. m. scutellata* and *A. m. capensis*.

## Discussion

“*I have just been thinking, and I have come to a very important decision.* These are the wrong sort of bees*.*”—Winnie the Pooh.

### A mitogenomic phylogeography of species of *Apis* and subspecies of *Apis mellifera*

The mitogenomic analysis confirms with high confidence previous inferences as to morpho-behavioral relationships among nine species as Dwarf, Giant, and Cavity-Nesting Honey Bees (Figs. 1a,b). Within the Apini, rooting with *Bombus* (Bombini) confirms Dwarf Honey Bees [*A. florea* + *A. andreniformis*] as outgroup to the Giant Honey Bees [*A. dorsata* + *A. laboriosa*] and the remaining, Cavity-Nesting Honey Bees. *A. mellifera* is the sister group to these [*A. koschevnikovi* + [*A. nigrocincta* + [*A. nuluensis* + *A. cerana*]]], at a greater phenetic distances than among any other *Apis* species pairs.

Mitogenomic sequences assigned to the taxon *A. m. mellifera* in GenBank occur in nine lineages collectively made paraphyletic by the placement of other subspecies. The phylogenetic analysis indicates that most of these apparent anomalies are artefacts of miss-assignment of individual sequences to the type subspecies. As noted, the Kenyan bee series when correctly referred to subspecies accords with the cladistic arrangement described here. The Arabian bee series referred to *A. m. mellifera* in GenBank falls phylogenetically into two distinct clades, one as part of the basal *A. m. mellifera* sensu lato and the other *A. m. jemenitica* sensu lato, along with Kenyan bees reassigned to the same subspecies. Another two Kenyan sequences assigned to *A. m. mellifera* are more closely related to *A. m. simensis* and *A. m. unicolor*, respectively, which suggests they are in fact members of those two subspecies. The latter extends the subspecies range beyond insular Malagasy.

### A revised phylogeographic hypothesis for the evolution of *A. mellifera*

The received picture of the evolution of *A. mellifera* has been an “Out of Africa” model^9^, with as many as three Recent excursions, originally to Europe, more recently to Iberia, and in historic times to South America as so-called “Killer” or “Africanized” Bees, which resulted from accidental crossing of imported *A. m. scutellata* queen with local *A. m. ligustica* drones^7^. This has been challenged more recently by models of “Out of Asia”^10^ or North African/Middle Eastern^12^ origins. The alternative phylogeographic clade structure presented here indicates that Things Fall Apart with any of these hypotheses. The mitogenomic phylogeography (Fig. 3) indicates instead that *A. mellifera* evolved as a North-to-South expansion from Europe to Africa by way of Asia Minor and the Levantine. The type form *A. m. mellifera* sensu stricto originated in northern Europe, and has diversified as *A. m. ligustica* in southeastern Europe, including expansion into Asia Minor (*A. m. caucasia*). European bees then spread southward via the Levant (*A. m. syriaca*) into Nilotic East Africa (*A. m. lamarckii*), and across the Red Sea into the Yemeni coast of the Arabian Peninsula (*A. m. jemenitica*). Southward expansion has left Ethiopian (*A. m. simensis*) and Malagasy (*A. m. unicolor*) lineages as earlier offshoots. Sub-Saharan mainland forms constitute a single clade that comprises individuals referred to *A. m. scutellata* and *A. m. capensis*, as well as *A. m. adansonii* and *A. m. monticola* both closely related to sequences assigned to the former and latter subspecies, respectively. *A. m. adansonii* occurs throughout central Africa: the single available sequence from Niger may not be representative. The structure of the Mediterranean clade indicates a secondary return to Europe during a refugial period, and a more recent tertiary return to North Africa via the western Mediterranean islands.

### Inferences from an mtDNA-based molecular clock for the evolution of *A. mellifera*

Divergence times within and among animal species, including Insecta, are routinely estimated from measured molecular divergences, including those made from mtDNA genomes^29^, including *Apis*^30^. Calibration of a molecular clock requires reliably dated external events, often geographic^31^, and accurate measurements of rates of substitution or divergence (2 × the former). Reliable geographic events are unavailable for *A. mellifera*^30^; substitutions per bp measured here over complete coding regions avoid gene-specific variance. Based on a fixed rate of 0.0115 substitutions/site/Myr (Brower 1994 in Ref.^31^) diversification of major lineages within *A. mellifera* can be dated to the Chibanian Age (late Pleistocene), 770–126 Kya. This coincides with and may be influenced by the European Günz glacial cycles. Separation of more southerly lineages from continental European subspecies occurred ca. 720 Kya, separation of African-endemic from Levantine/Nilotic/Arabian lineages ca. 660 Kya, establishment in Africa ca. 540 Kya, and separation of Euro-African Mediterranean from Sub-Saharan lineages ca. 250 Kya. Diversity within several subspecies dates to > 100 Kya, notably among multiple replicates of *A. m. ligustica* and *A. m. jemenitica*, 120 Ka and 150 Ka, respectively. Differences among other nominal subspecies originate only a few 10 s of Kya, notably the Asia Minor forms (*A. m. meda*, *A. m. caucasia*, and *A. m. anatoliaca*, 10–20 Ka) and Mediterranean species including insular forms (*A. m. ruttneri* and *A. m. siciliana*, 40 Kya).

### Previous inferences from morphology

**Figure 5:**
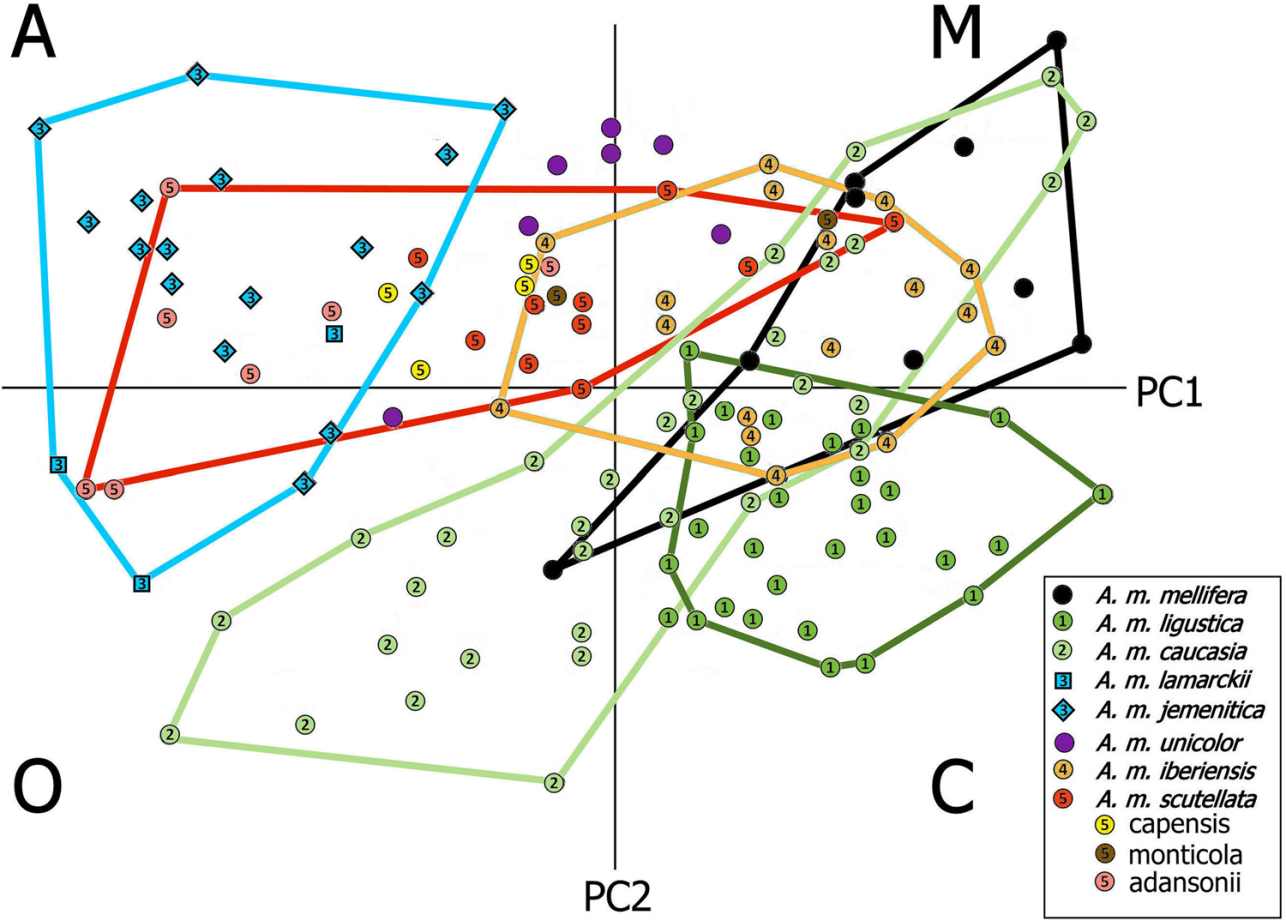
Molecular clades of *A. mellifera* mapped onto the first two PCA axes of morphometric **ACMO** space, redrawn after Fig. 10.8 in Ref.^8^. Individual bees are identified to subspecies by the numeric color codes, and re-grouped to genetic clades as in Fig. 3, with additional color variants for Sub-Saharan taxa. Ruttner’s four groups [African (**A**), European (**C** [Continental]), Mellifera (**M**), and Asian (**O** [Oriental]] correspond roughly to the four quadrants, clockwise from the upper left as **A**, **M**, **C**, and **O**. The Levantine/Nilotic/Arabian  clade overlays the Sub-Saharan clade , including local variants. The Asia Minor *A. m. caucasia* clade  is distributed along the **M → O** axis, and is bisected by its Southeast European sister clade *A. m. ligustica*. The Afro-European Mediterranean clade  overlies all three, as well as the basal *A. m. mellifera* clade.

The standard classificatory and evolutionary system of *Apis* is based on meristic analysis of four continental groups, African (**A**), European (**C**), Mellifera (**M**), and Asian (**O**)^8^. In Fig. 5 (redrawn from Fig. 10.8 in Ref.^8^), the placement of individual bees in Principal Components space corresponds roughly to the four quadrants of the first two axes, clockwise from the upper left as **A**, **M**, **C**, and **O**, respectively. Re-tagged and grouped genetically, the Arabian/Nilotic clade overlies the Sub-Saharan clade, including local variants. However, the Asia Minor *A. m. caucasia* clade is distributed along the **M → O** axis, and is bisected by its Southeast Europe sister clade *A. m. ligustica*. Although the African and European/Asian clades are essentially non-overlapping, the Mediterranean clade overlies all three, as well as the basal *A. m. mellifera* clade. Despite their dispersion in PC space, *A. m. caucasia* and *A. m. iberiensis* are the least genetically diverse clades; individuals in the latter plot are almost entirely African. Consolidation of all “African” subspecies as a single group does not recognize the origins of the Euro-African-Mediterranean subspecies, nor does a single “Asian” group recognize the complex biogeographic connection of Eurasian subspecies in southeast Europe and Asia Minor, or of these with Africa via the Levant.

The **ACMO** model is taken as support for an argument against an Oligocene/early Miocene dispersal from Europe to Africa. More recent morphological analysis of wing-venation patterns including fossil material^32,33^ suggests a European origin of *A. mellifera* antecedents from an *A. cerana*/*dorsata* type in the Miocene, in agreement with the molecular clock estimate here. However, the hypothesized evolution of the *A. mellifera* type in Sub-Saharan Africa late in the epoch is according to the clock too far back, and return to Europe in the Holocene is both too far forward and in the opposite direction. Despite European antecedents, this remains a “(Long) Out of Africa” model. The similarity dendrogram in Fig. 1 of Ref.^32^ shows [*Bombus* + [[*A. dorsata* + [*A. florea* + [*A. cerana* / *A. mellifera*]]]]] with *cerana* & *mellifera* forms co-mingled. This arrangement transposes *A. florea* and *A. dorsata* and therefore the Dwarf and Giant Honey Bee morphotypes (cf. Fig. 1a), and fails to resolve the deep phylogenetic separation of *A. cerana* / *A. mellifera* (cf. Fig. 1b and Supplementary Fig. S4).

### Previous inferences from whole mitogenomes

**Figure 6:**
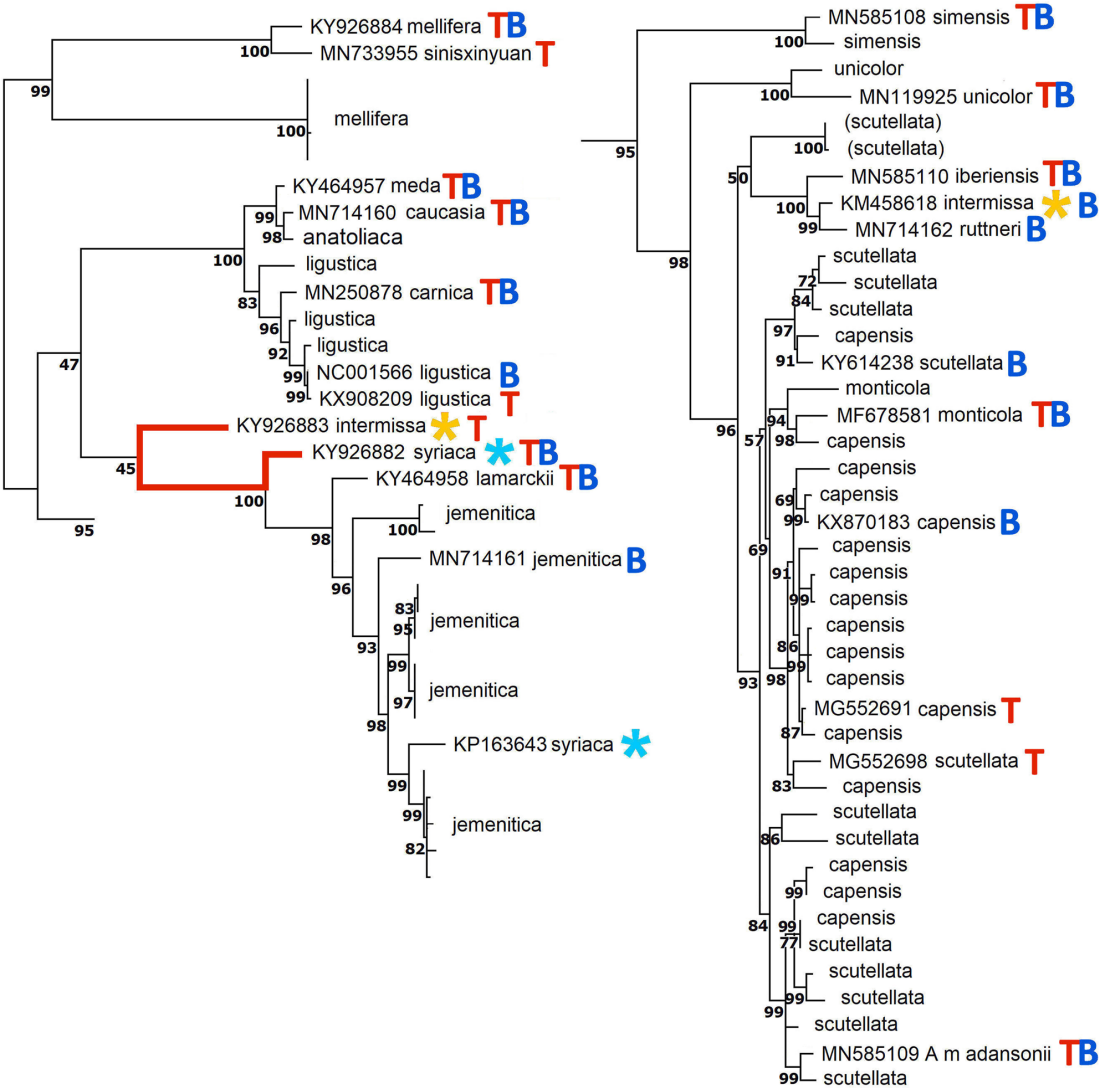
Comparison of Boardman et al.^11^ and Tihelka et al.^20^ meta-analyses with Fig. 2. The base phylogram is a Maximum Parsimony analysis calculated as in Fig. 2, with the addition of two problematic sequences mentioned in the text, KY926882 and KY926883, attributed to *A. m. syriaca* and *A. m. intermissa*, respectively. The 17 sequences used by Boardman et al. are marked “” and the 16 sequences used by Tihelka et al. “”, with the 11 sequences common to both sets “”. Note the anomalous pairwise placements of two sequences attributed to KY026882 and KY926883 with respect to their nominal sister subspecies ( and , respectively).

Previous meta-analyses by Boardman and Eimanifar et al.^11,16–22,25,26^ and Tihelka et al.^12^ are compared with the present analysis in Fig. 6. The base phylogram is a Maximum Parsimony analysis calculated as in Fig. 2, with the addition of two problematic sequences noted in the text, KY926882 and KY926883, attributed to *A. m. syriaca* and *A. m. intermissa*, respectively. KY926882 “” falls outside the *jemenitica* cluster that includes *A. m. syriaca* KP163643, which is not used in either the “” or “” sets. KY936883 “” is more closely related to KY936882 than to *A. m. intermissa* KM458618, which occurs where predicted biogeographically. There are indications of faulty data in KY936882 and KY936883: inspection of the last quarter of the aligned sequences shows two unbroken runs of seven shared phylogenetically informative sites^31^ that unite KY936882 and KY936883 to the exclusion of their nominal subspecific sisters. In the Sub-Saharan clade, the *A. m. capensis*/*A. m. scutellata* pair (KX870183/KY614238) in the “” set occurs in *A. m. capensis* + *A. m. scutellata* clade **I** (but not **II**), and in the “” set MG552698 appears to be mis-referred to *A. m. scutellata* instead of *capensis*. *A. m. adansonii* (MN585109) “” is more closely related to the other, larger clade of *A. m. scutellata*. *A. m. monticola* (MF678581) “” is more closely related to *A. m. capensis*. Thus, reliance on single, atypical sequences in previous meta-analyses has led to faulty phylogenetic inferences.

Boardman, Eimanifar et al. [^11^ *et seq*.] root their species-inclusive trees at the midpoint, rather than by an outgroup, which as drawn implies that the Dwarf and Giant Honey Bee species are sister groups, rather than that the former are the outgroup to the remaining species (cf. Fig. 1b). Their alignments include rDNA along with Coding Region sequences, which may contribute to the relatively weaker bootstrap support for some nodes: among subspecies of *A. mellifera*, my trial rDNA alignments were problematic at best, and these regions were excluded from the analysis here.

Tihelka et al.^12^ inferred their phylogeny by a BI site-heterogeneous mixture model. The root with respect to *A. cerana* falls between *A. m. intermissa* and other African (**A**) subspecies (notably *A. m. scutellata*), in support of an African origin, in contrast to Figs. 2 and 3. *A. m. mellifera* and *A. m. iberiensis* (**M**) are on the same side of the root as *A. m. intermissa*. The Asian (**O**) subspecies are further along the backbone, followed by the European (**C**) subspecies. The Asian form indicated by “**?**” is unidentified: the likeliest candidate seems to be *A. m. caucasia*. Of 11 nodes in their tree, only the **C** group is found in Fig. 2 here, and none corresponding to groups **A**, **M**, **O**, or any of the three-member clades. Exclusion of third-positions SNPs in their P12 model yields essentially the inverse of the trees in Fig. 2, with African lineages first derived, and *A. m. mellifera* as outgroup to the Eurasian lineages. Their model transposes the African and Eurasian lineages to either side of *A. m. mellifera*. At least two nearest-neighbor subspecies pairs are markedly different: East African *A. m. simensis*/*A. m. unicolor*, *A. m. intermissa* with respect to *A. m. sahariensis*/*A. m. iberiensis*. Relationships among subspecies lineages are similar to those in Fig. 2 (equivalent to the P123 model), but with much lower bootstrap support. However, within the *A. m. simensis*-inclusive clade, structure and support for the African and Mediterranean lineages collapse in the P12 with respect to the P123 model, and bootstrap support for key branches is < 50%. Especially, MN119925 *A. m. unicolor* is transposed as a long branch from outside to inside these clades. Of course, not all first-position SNPs are substitutions, nor all third-position SNPs silent. A Maximum Parsimony analysis of inferred amino acid substitutions at 125 sites across representatives of 22 subspecies (Supplementary Fig. S5) gives a clade structure is similar to Fig. 2 here or that from their P12 model (except as to rooting), with bootstrap support for African and Mediterranean lineages again < 50%. Bootstrap support for other clades in Fig. 2 is strong.

As shown by the molecular clock in Fig. 4, pairwise differences among nominal subspecies within the Southeast European, Asia Minor, and Mediterranean clades are less than those between individuals within other subspecies, even where within-clade relationships are well-defined. Where these local subspecies were originally defined by perceived morphological differences, review of their alpha and beta taxonomy may be indicated, with a view towards synonymizing local forms in gamma taxonomy: names matter^27^.

### Additional phylogeographic inferences from partial mitogenome sequences

Besides the 22 subspecies of *A. mellifera* with complete mitogenomes compared in the main MS, GenBank includes six additional subspecies with partial sequences from the 5′ end of the ND2 region. Their names and provenance are *A. m. adami* (Crete), *A. m. cecropia* (Greece), *A. m. cypria* (Cyprus), *A. m. macedonica* (Greece into Romania), *A. m. pomonella* (Kazakhstan), and *A. m. sicula* (Sicily). Over the first 574 bp common to all these subspecies, in combination with the 22 subspecies’ mitogenomes examined in the main MS, there are 39 variable sites of which 19 are phylogenetically informative sensu Nei^34^ (Supplementary Fig. S6).

Sequences from subspecies *A. m. adami*, *A. m. cecropia*, and *A. m. macedonica* are identical to those from *A. m. ligustica* and *carnica* in the Southeast Europe clade, as is the sequence from *A. m. cypria* except for a single autapomorphic Y/G SNP. The sequence from *A. m. pomonella* is identical to that of *A. m. meda* in the Asia Minor clade. The sequence from *A. m. sicula* is identical with that of the reference *A. m. mellifera*, except for a T SNP otherwise found only in the African superclade.

Inclusion of these short sequences tests the phylogeographic hypothesis. The similarity of the Central Asian *A. m. pomonella* and Middle Eastern *A. m. meda* extends the Asia Minor clade further eastward, separately from *A. m. sinisxinyuan*. *A. m. sicula* is cladisitically distinct from *A. m. siciliana* in the Mediterranean clade, and the co-occurrence of the two forms on Sicily suggests a secondary colonization from the westerly islands after in initial occupation from the European mainland. Likewise, the continental association of *A. m. adami* and *A. m. cypria* from Crete and Cyprus, respectively, is consistent with a dispersal corridor from southeastern Europe through the eastern Mediterranean and into the Levant, and suggests an eastern limit of the re-colonization of the insular Mediterranean at Sicily. The phylogeography proposed here accommodates these data well: further insight may be expected from complete mitogenomes.

### Previous inferences from nucDNA genome sequences

The “*Thrice Out of Africa*” hypothesis^9^ follows a phylogenetic scheme that retrieved the same four groups (**ACMO**) as the then current continental geographic scheme based on meristics and morphology^8^. Their molecular network transposes the alphabetical order to an **MAOC** backbone, rooted with respect to a composite outgroup within the (**A**)frican subspecies, which along with Mellifera (**M**) was separated from the European (**C**ontinental) and Asian (**O**riental) subspecies. Subsequent analyses of various nucDNA markers and combinations of *A. mellifera* subspecies broadly identify the **MAOC** groups^9,10,23,24,32,33,35^, however placement of the root on various branches according to internal and (or) external criteria varies, so as to suggest alternative geographic origins of *A. mellifera*. These include alternative interpretations of the Thrice Out of Africa data^29^ [root indeterminate between **M + A** and **O + C**], as well as Thrice Out of Asia^9^ and North African/Middle Eastern origin models^10,32,33,35^, for example with *A. m. jemenitica* in the **Y** lineage as outgroup to other subspecies of **A**.

There are fundamental differences between the **MAOC** backbone and mtDNA genome phylogenies (Supplementary Fig. S7), both with respect to placement of the root and allocation of subspecies to clades. No **MAOC** model places *A. m. mellifera* as outgroup to other subspecies: the mtDNA data place *A. m. scutellata* distal rather than proximal to the root of *A. mellifera* evolution. Closely related subspecies and geographically contiguous subspecies within the Mediterranean mtDNA clade here, including *A. m. iberiensis* and *A. m. intermissa*^9,32^, and *A. m. ruttneri*^24^, are dispersed over the **M**, **A**, and **C** clusters, respectively. The *A. m. jemenitica*/*A. m. lamarckii*/*A. m. syriaca* clade here is also dispersed over the **A**(**Y**), **A**(**L**), and **O** clusters. On the other hand, pairings of *A. m. ligustica* and *A. m. carnica* (**C**) and *A. m. anatoliaca* and *A. m. caucasia* (**O**) are consistent between nuc- and mtDNA data. I note that allocation of subspecies among rooted **MAOC** clusters conforms closely to the original continental model^8^, which was not cladistic in approach or form. The mtDNA-based model explains a more complex continental distribution, where European, Asian, and African subspecies are of multiple origin.

Classification of nucDNA SNP markers from eight of the subspecies examined here^24^ *inter alia* overlays *A. m. anatoliaca* and *A. m. caucasia* in the **O** group, and separates *A. m. iberiensis* from *A. m. mellifera* (three clusters) in the **M** lineage. *A. m. ruttneri*, the only representative of the African **A** group, is placed midway between *A. m. mellifera* and the **C** group, rather than close to *A. m. intermissa* and *A. m. iberiensis* in the Mediterranean clade as here. That is, lineages paired in the two subspecies of the Eurasian clade and variants within the Mediterranean clade here are separated by SNP data in contrast to the clade hierarchy here.

Contrasts between nucDNA, mtDNA, and even morphological phylogenies are not unknown^29^. Maternally-inherited mtDNA phylogenies have the virtue of tracing maternal evolutionary lineages^36^, and may thus be particularly reliable for phylogeographic inferences about the origins and spread of “queen”-dispersed eusocial insect. A phylogeographic origin Out of Europe inferred from prior affinity with central Asian cavity-nesting bees seems more parsimonious that disjunct Sub-Saharan or Asian origins.

## Conclusions

1. Rooting of complete mtDNA Coding Region sequences from eight species of *Apis* (Apini) with that of a bumble bee (*Bombus ignitus*: Bombini) confirms a Miocene origin of Dwarf Honey Bees as outgroup to the Giant and Cavity-Nesting species groups, the latter including the Western Honey Bee (*A. mellifera*).
2. Phylogenetic analysis that includes multiple mtDNA genomes sequences per subspecies indicates that the Western Honey Bee originated in Northern Europe during the late Pleistocene. The phylogeographic distribution of its component subspecies clades indicates that Things Fall Apart with either the Out of Africa or Out of Asia hypotheses, when those are based on single mitogenomes per subspecies.
3. From a basal origin in Northern Europe, *A. mellifera* dispersed to Southeast Europe (*A. m. ligustica*/*A*. *m. carnica*/*carpatica*) and extended to Asia Minor (*A. m. meda*/*A. m. caucasia*/*A. m. anatoliaca*). The European lineage then spread southward via the Levant into the Nile Valley, East Africa and Arabia (*A. m. syriaca*/*A. m. lamarckii*/*A. m. jemenitica*), and thence into sub-Saharan Africa (*A. m. simensis*/*A. m. unicolor*; paraphyletic *A. m. scutellata* & *A. m. capensis* including *A. m. adansonii* and *A. m. monticola*). The Mediterranean lineage was re-established from an African lineage in Iberia and the western Mediterranean islands (*A. m. iberiensis*/*A. m. ruttneri* + *A. m. siciliana*/…), and thence spread back into North Africa (…/*A. m. sahariensis* + *A. m. intermissa*). Partial sequences from more easterly insular Mediterranean subspecies suggest affinity with the Mellifera (*A. m. sicula*, Sicily) or Southeast European lineages (*A. m. cyprii*, Cyprus; *A. m. adami*, Crete).
4. A molecular clock estimates the European origin of *A. mellifera* ca. 780 Kya, separation of more southerly lineages ca. 720 Kya, separation of African from Levantine/Nilotic/Arabian lineages ca. 660 Kya, spread to Africa ca. 550 Kya, and separation of Mediterranean from Sub-Saharan lineages ca. 250 Kya. Diversities within several subspecies date to > 100 Kya, whereas difference between Asia Minor and insular Mediterranean subspecies date to only a few 10 s of Kya.
5. Inclusion of multiple sequences referred to *A. m. mellifera* in GenBank shows that apparent paraphyletic anomalies within and among subspecies are in several cases artefacts of mis-assignment to the type subspecies. Incorrect phylogenetic inferences may also proceed from use of non-representative or faulty sequences.
6. Morphology- and (or) nucDNA-based biogeographic models are not consistent with this multi-mitogenome per taxon model. Genetic differences between nominal southeast European, Asia Minor, and Mediterranean subspecies are typically much less than those within other subspecies for which multiple individual sequences are available. Re-consideration of the alpha and gamma taxonomy of these taxa is indicated. Names matter.

## Supplementary Information

Supplementary Information 1.

Supplementary Information 2.

Supplementary Information 3.

Supplementary Information 4.

Supplementary Information 5.

Supplementary Information 6.

Supplementary Information 7.

Supplementary Information 8.

## Data Availability

All mtDNA genome sequences used were obtained from and are available through NCBI GenBank. The accession numbers of the 66 *Apis mellifera* ssp sequences analyzed in detail are given in Table 1 and Supplementary Figs. S1–S3; 12 additional accessions referred to in Fig. 2 are also given there, and two questionable sequences are given in Fig. 6. Accession numbers of the sequences of nine species of *Apis* examined and the *Bombus ignitus* outgroup are given in Fig. 1a.
