# Supplementary material for: Multiple mitogenomes indicate Things Fall Apart with Out of Africa or Asia hypotheses for the phylogeographic evolution of Honey Bees (Apis mellifera)
Source: Sci Rep. 2023 Jun 9;13:9386. doi: 10.1038/s41598-023-35937-4 (PMC10256785; doi:10.1038/s41598-023-35937-4)
Supplement: Supplementary file 1 — Supplementary Information 1. [file 41598_2023_35937_MOESM1_ESM.docx]

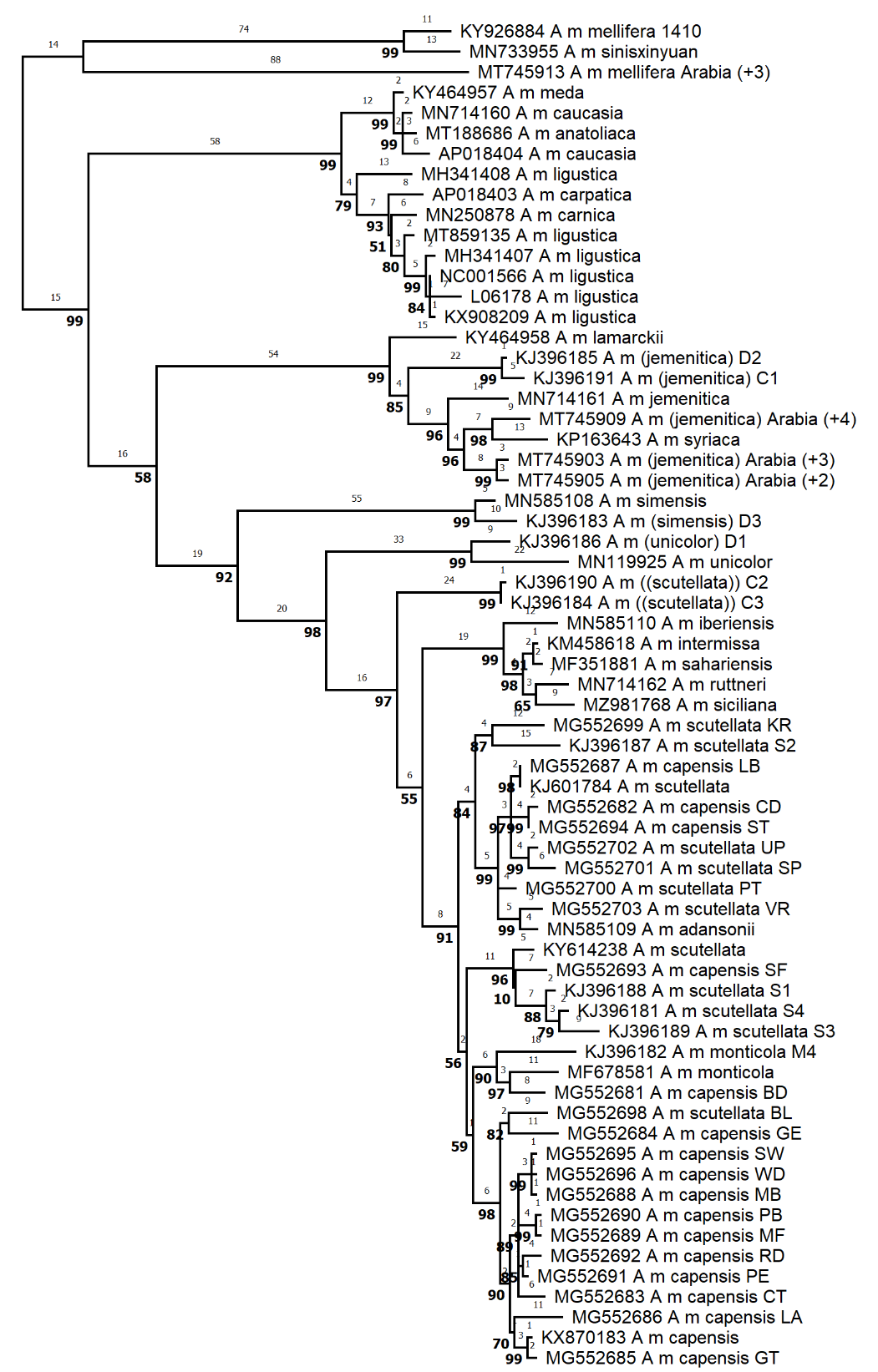


Supplementary Figure S1 – Maximum Parsimony analysis of phylogenetic relationships among 78 individual *A. mellifera*. Individuals are the 66 included in Figure 2, identified by GenBank accession numbers, plus 12 individual Arabian bees identical to those shown.


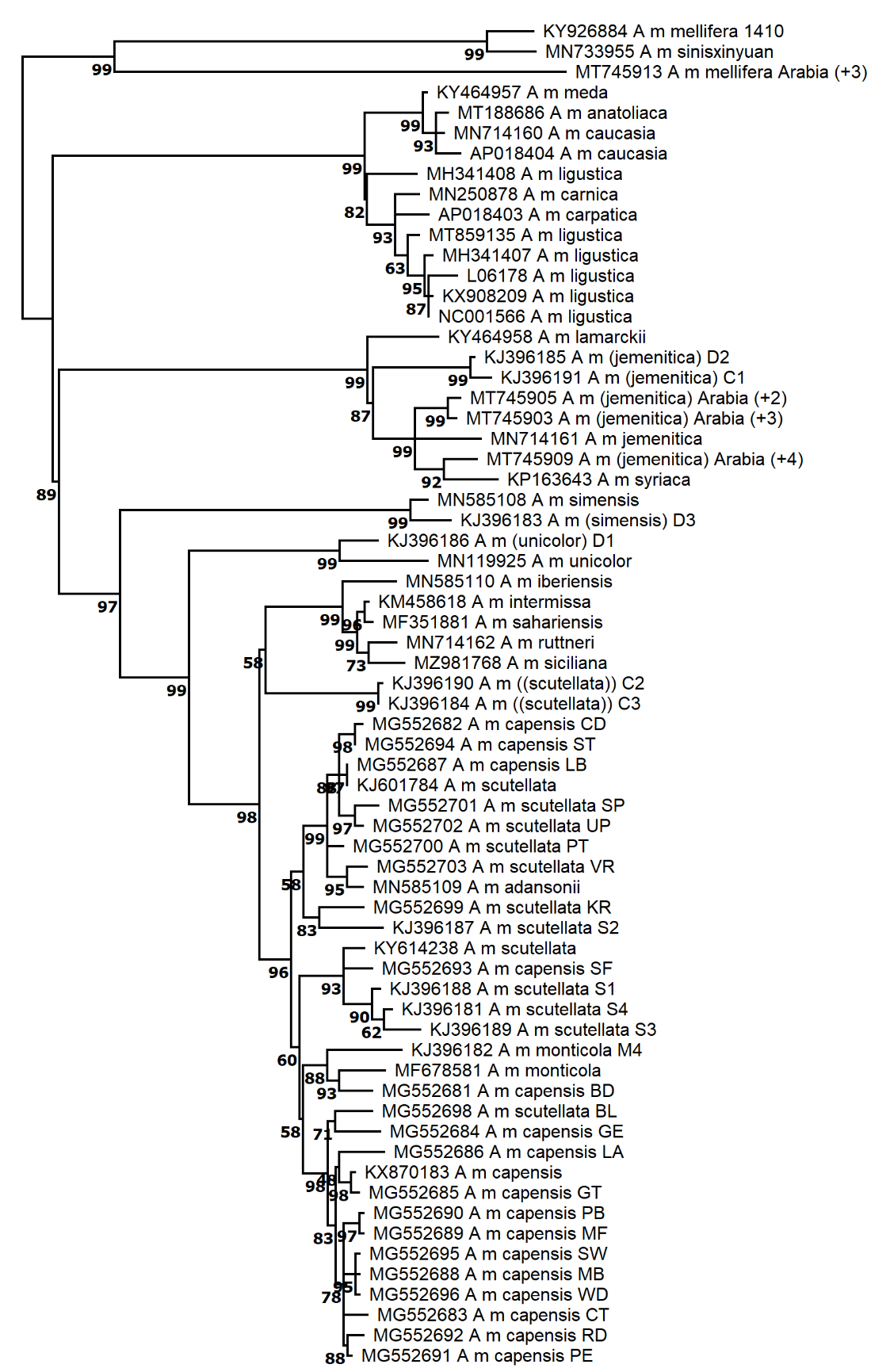


Supplementary Figure S2 – Maximum Likelihood analysis, as in Supplementary Figure S1.


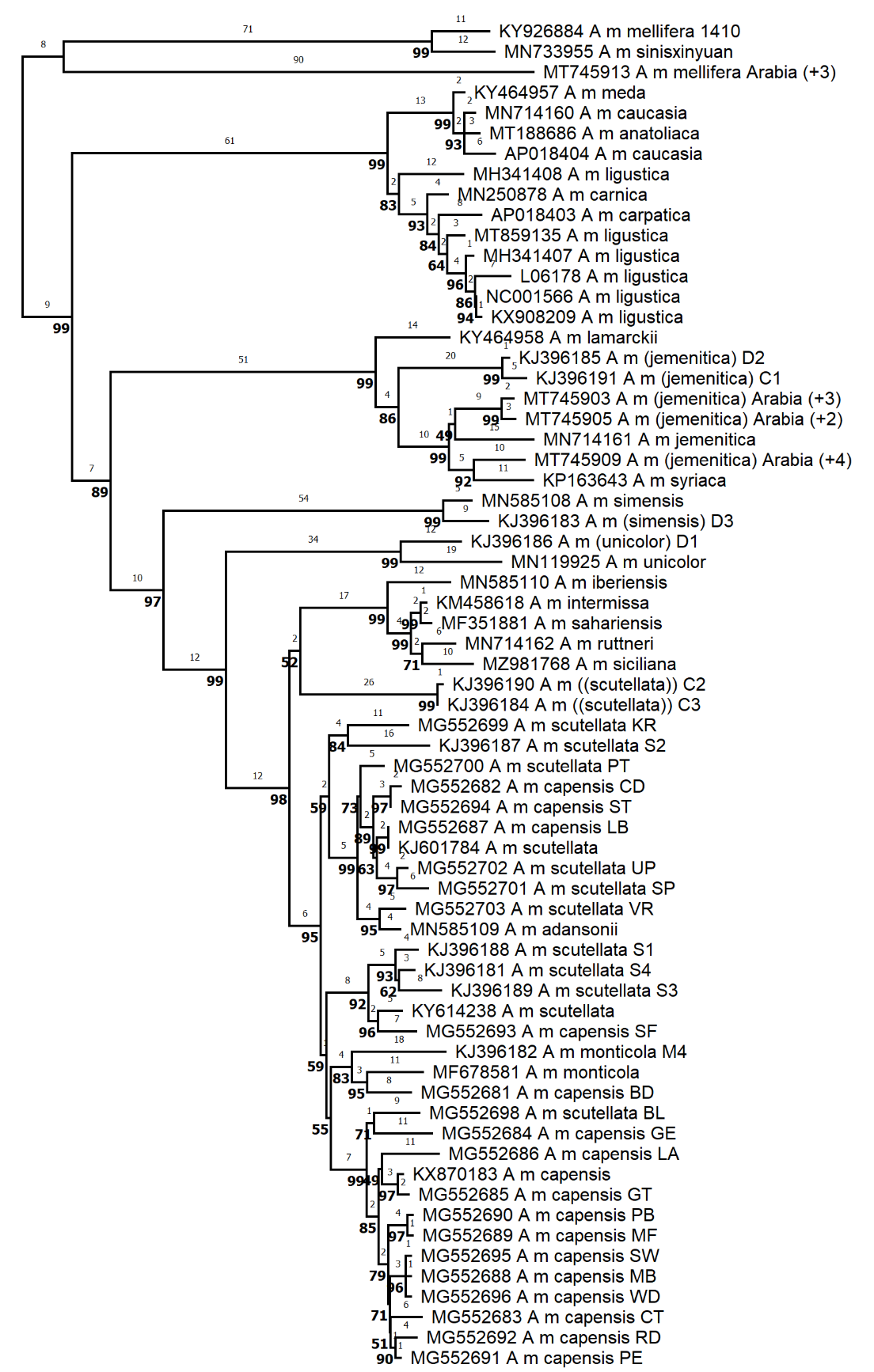


Supplementary Figure S3 – Neighbor-Joining analysis, as in Supplementary Figure S1.


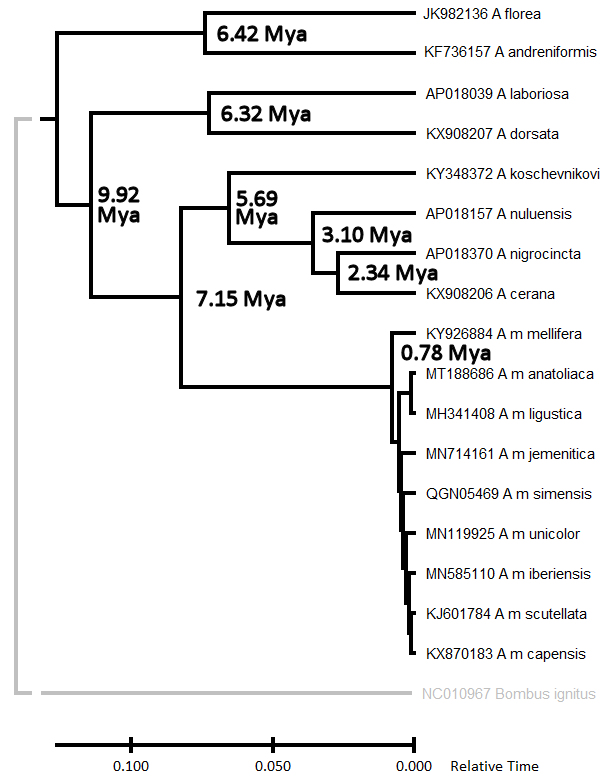


Supplementary Figure S4 - An mtDNA-based molecular clock for species of *Apis*. Divergence times are calculated as in Figure 4, with *Bombus* as outgroup (cf. Figure 1).


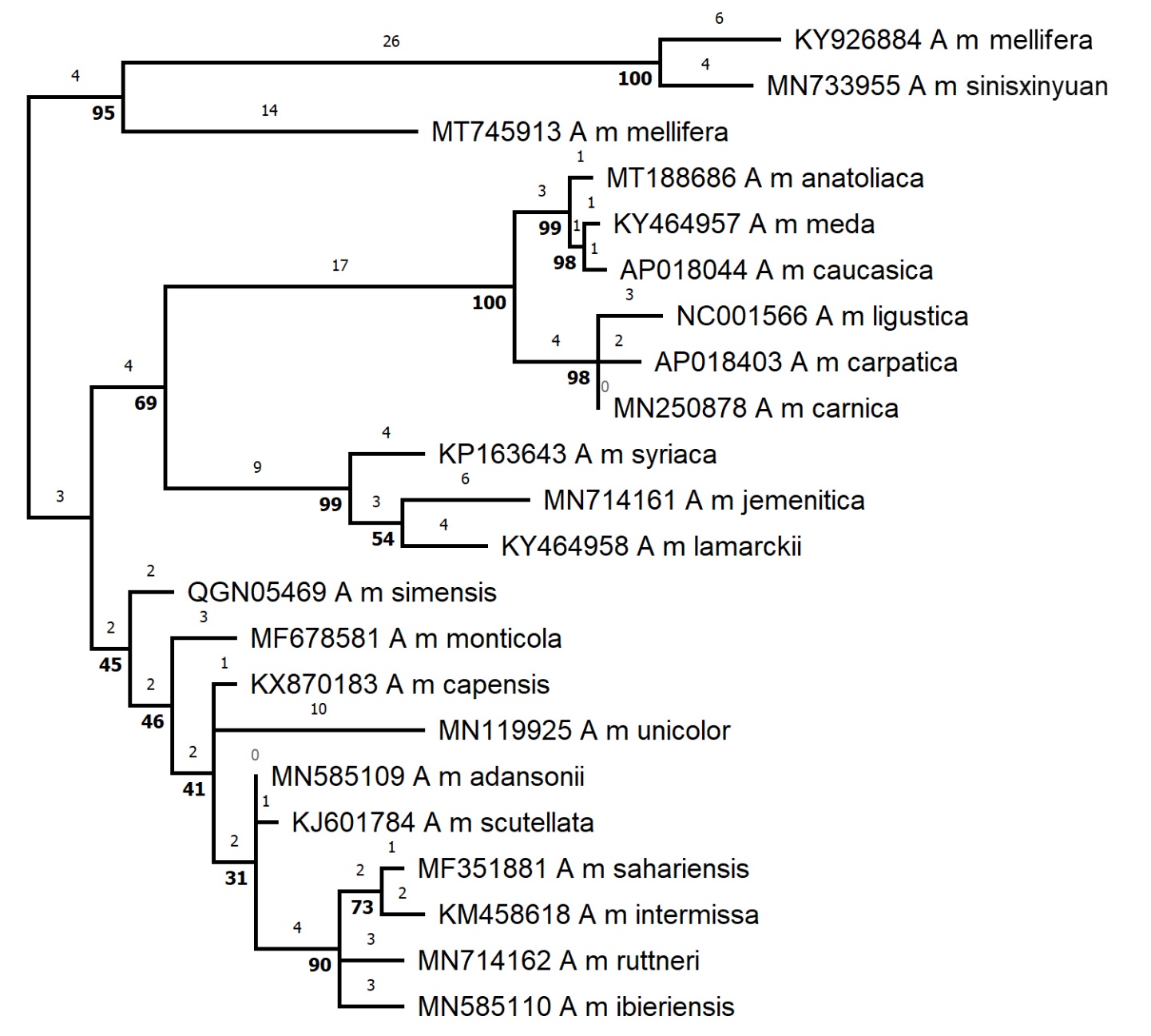


Supplementary Figure S5 - Maximum Parsimony analysis of 125 amino acid variants across representatives of 22 subspecies of *A. mellifera*. Numbers above each branch are the inferred numbers of amino acid substitutions; numbers below each node are the support among 3,000 bootstrap replications. Note that support for most nodes among African subspecies are < 50%.


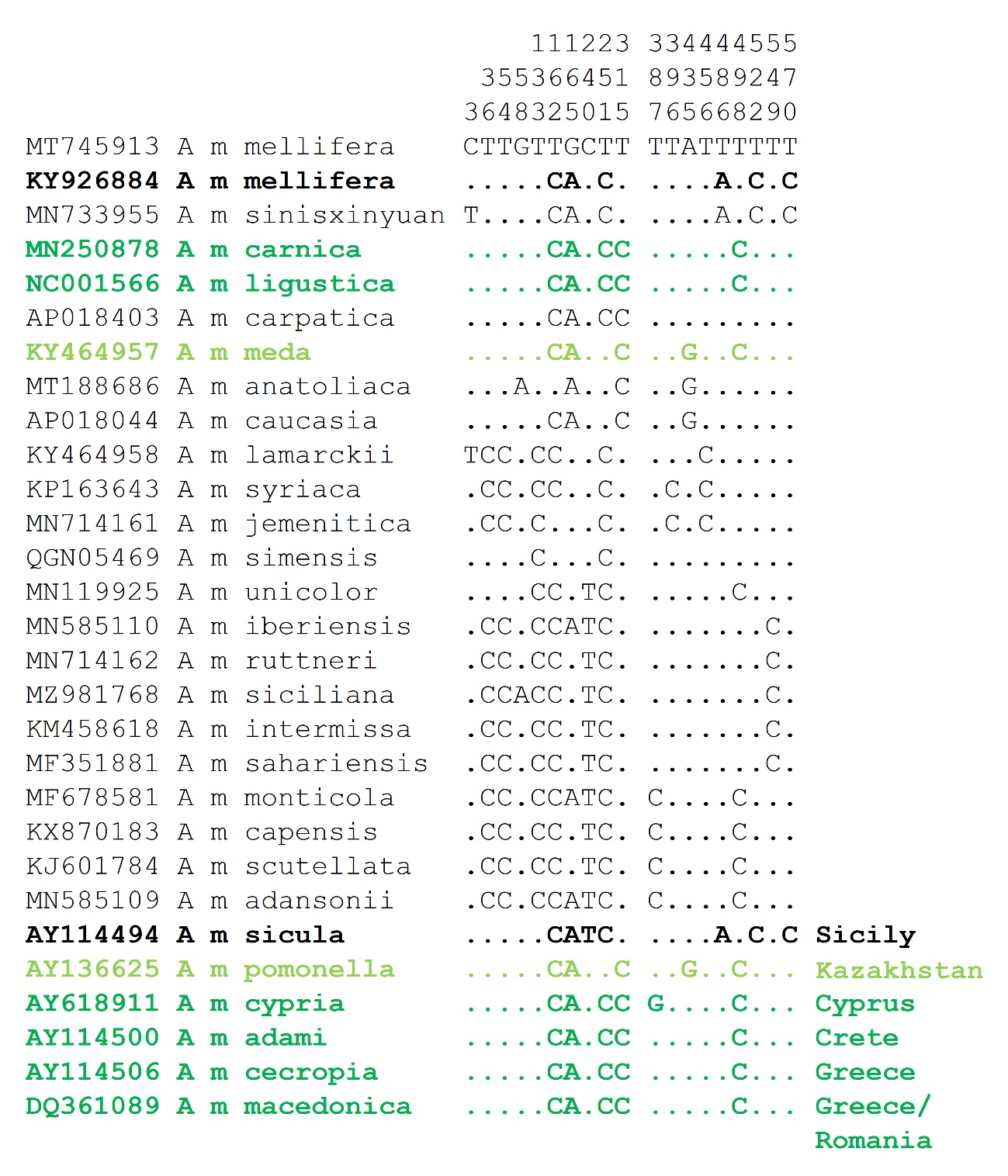


Supplementary Figure S6 – Phylogenetically Informative Sites in the 5’-most 574 bp of the ND2 coding region for 28 subspecies of *A. mellifera*. The last six sequences are in addition to those analyzed in the main text. Sequences in dark green are shared with the SE Europe clade, in light green with the Asia Minor clade, and in black with the reference *A. m. mellifera* sequence. Positions are numbered from #1 in the 11,006 Coding Region alignment.


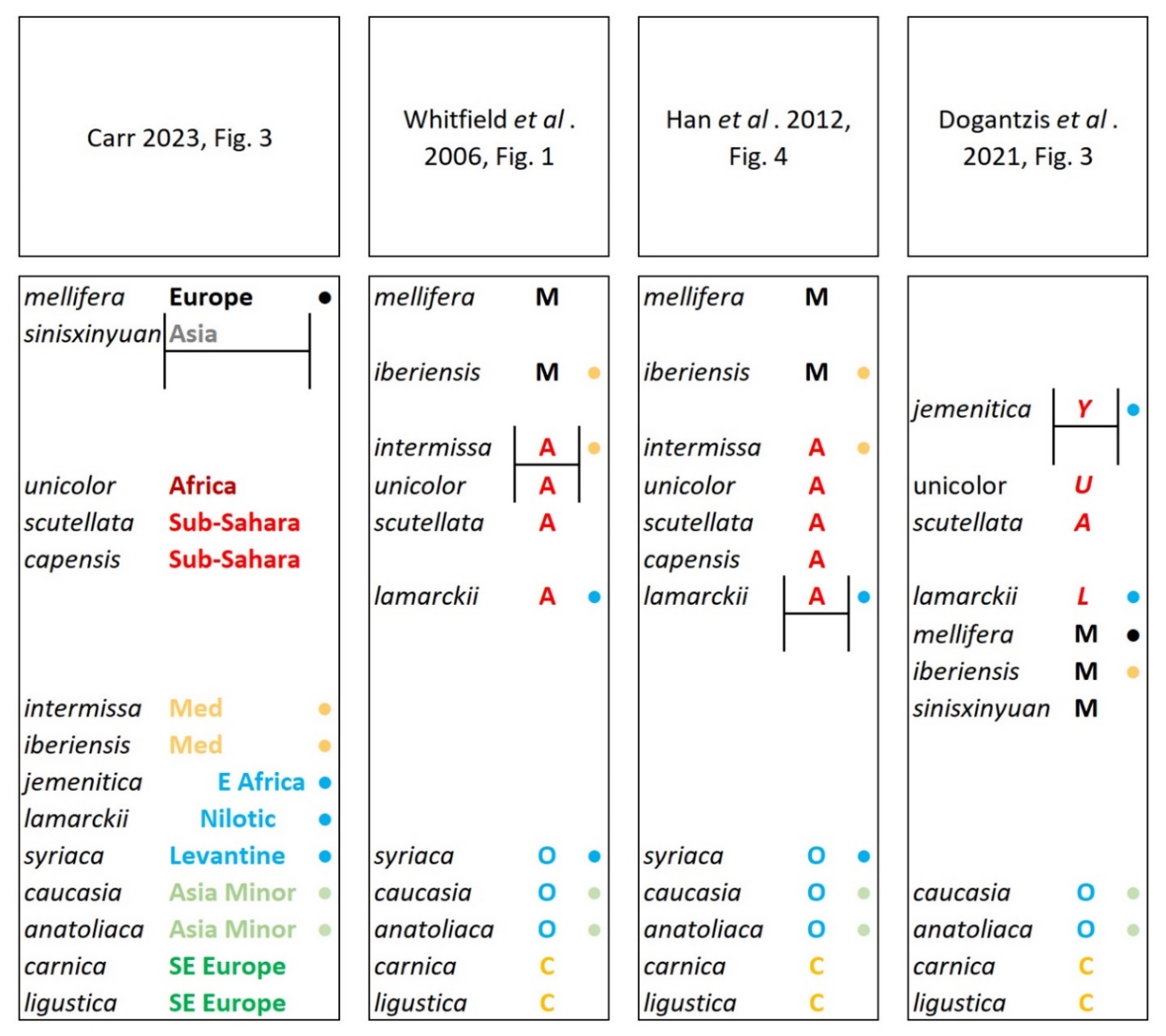


Supplementary Figure S7 – Schematic comparison of mtDNA genome phylogeny *versus* nucDNA phylogenies of *A. mellifera*. Order of taxa is based on a linearized ordering of **MAOC** as in Whitfield *et al*. (2006)^4^: placement of the root is indicated by **|---|** between the basal-most clades. MtDNA color codes as in Figure 2; nucDNA color codes for **M**ellifera, **A**frican, **O**riental [Asian], and **C**ontinental [European] clusters as in the referenced MSS^4,25,7^. Comparative placement of taxa as inferred from mtDNA *versus* nucDNA data are indicated by color-coded ●’s, referenced to Figure 2: see main text. *A. m. ruttneri* (not shown) is in the Mediterranean mtDNA clade and the **O** nucDNA cluster^19^.
