# Supplementary figures and images for: Multiple mitogenomes indicate Things Fall Apart with Out of Africa or Asia hypotheses for the phylogeographic evolution of Honey Bees (Apis mellifera)
Source: Sci Rep. 2023 Jun 9;13:9386. doi: 10.1038/s41598-023-35937-4 (PMC10256785; doi:10.1038/s41598-023-35937-4)

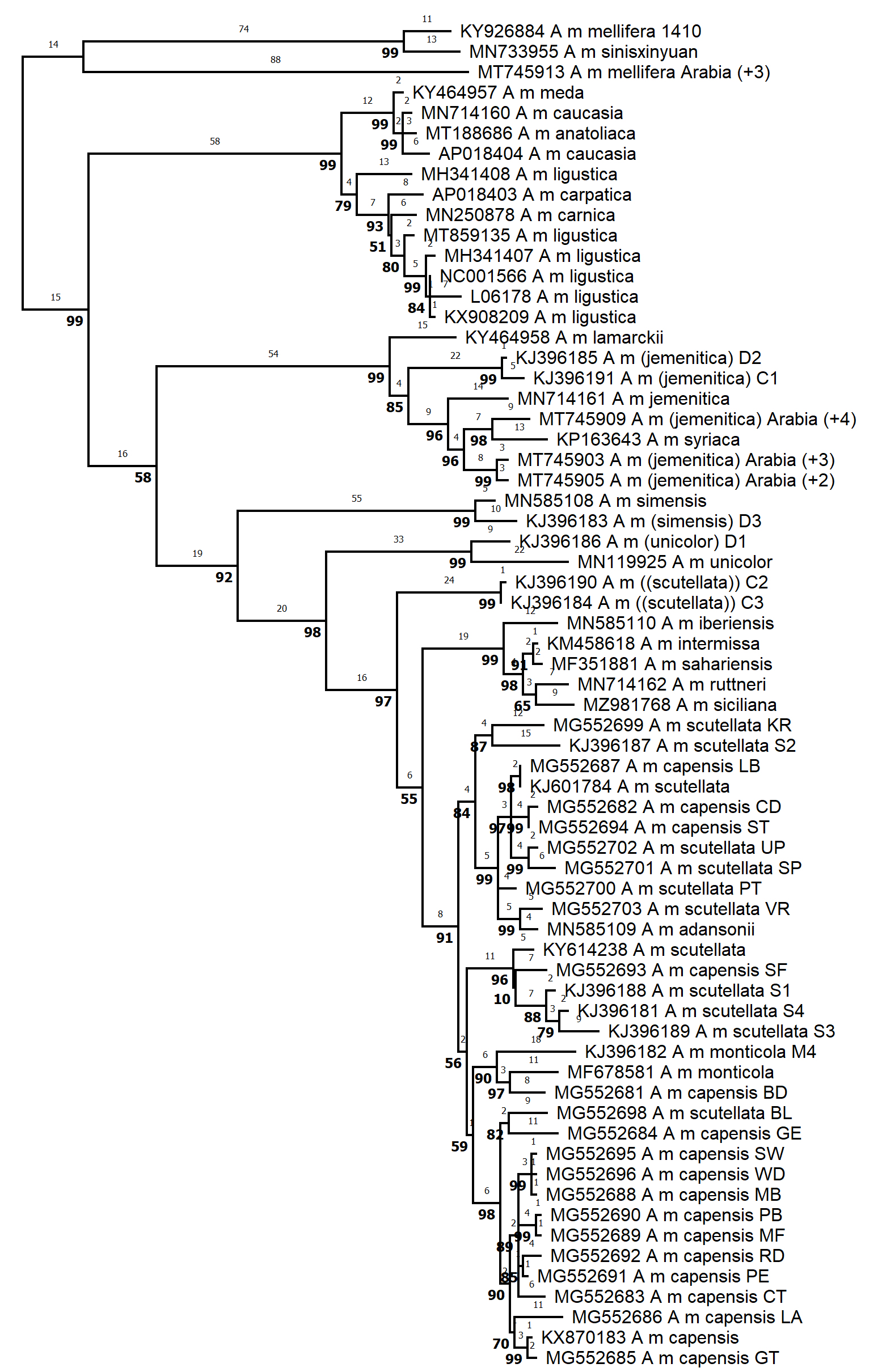

Supplement: Supplementary file 2 — Supplementary Information 2. [file 41598_2023_35937_MOESM2_ESM.jpg]

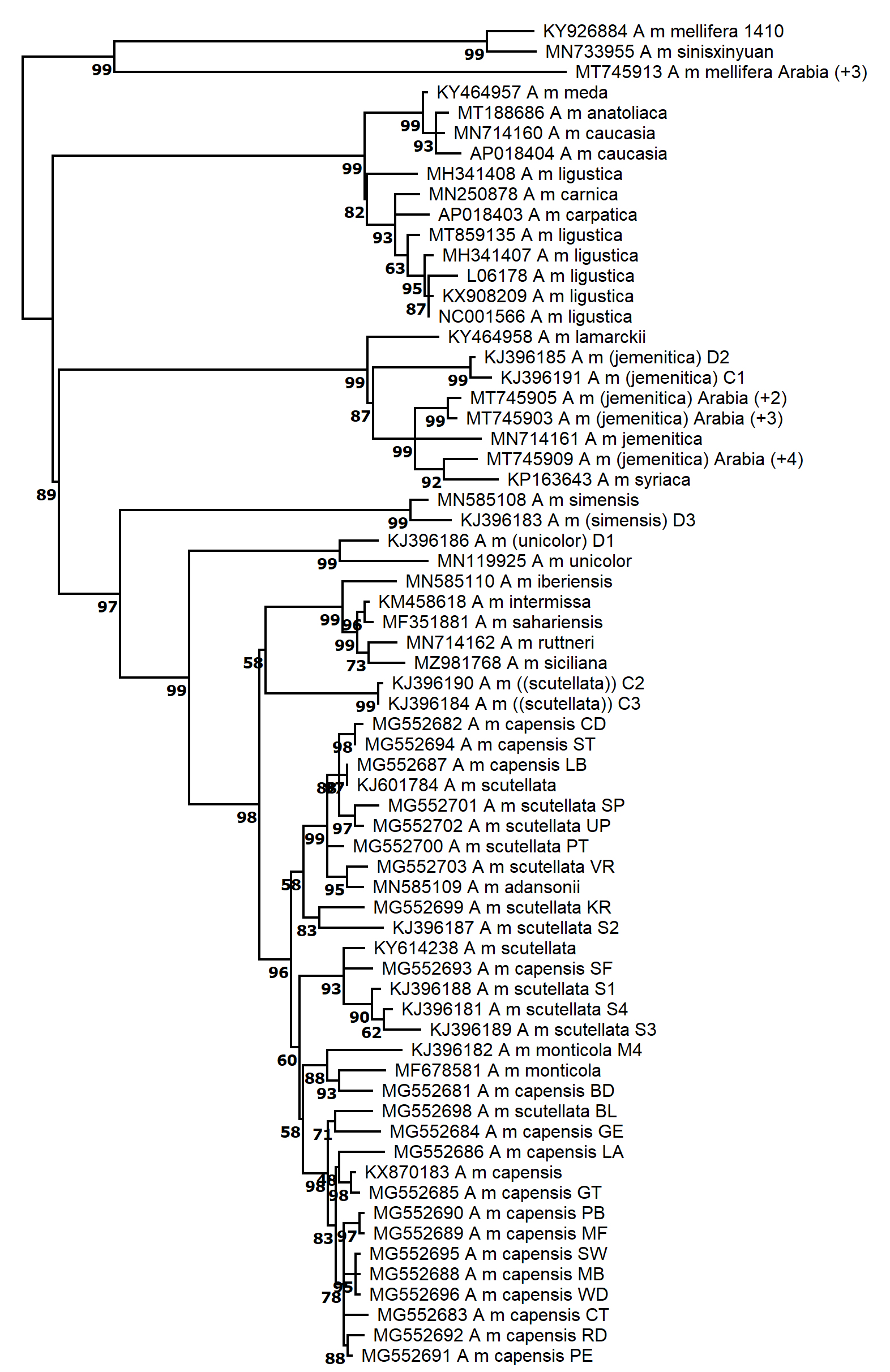

Supplement: Supplementary file 3 — Supplementary Information 3. [file 41598_2023_35937_MOESM3_ESM.jpg]

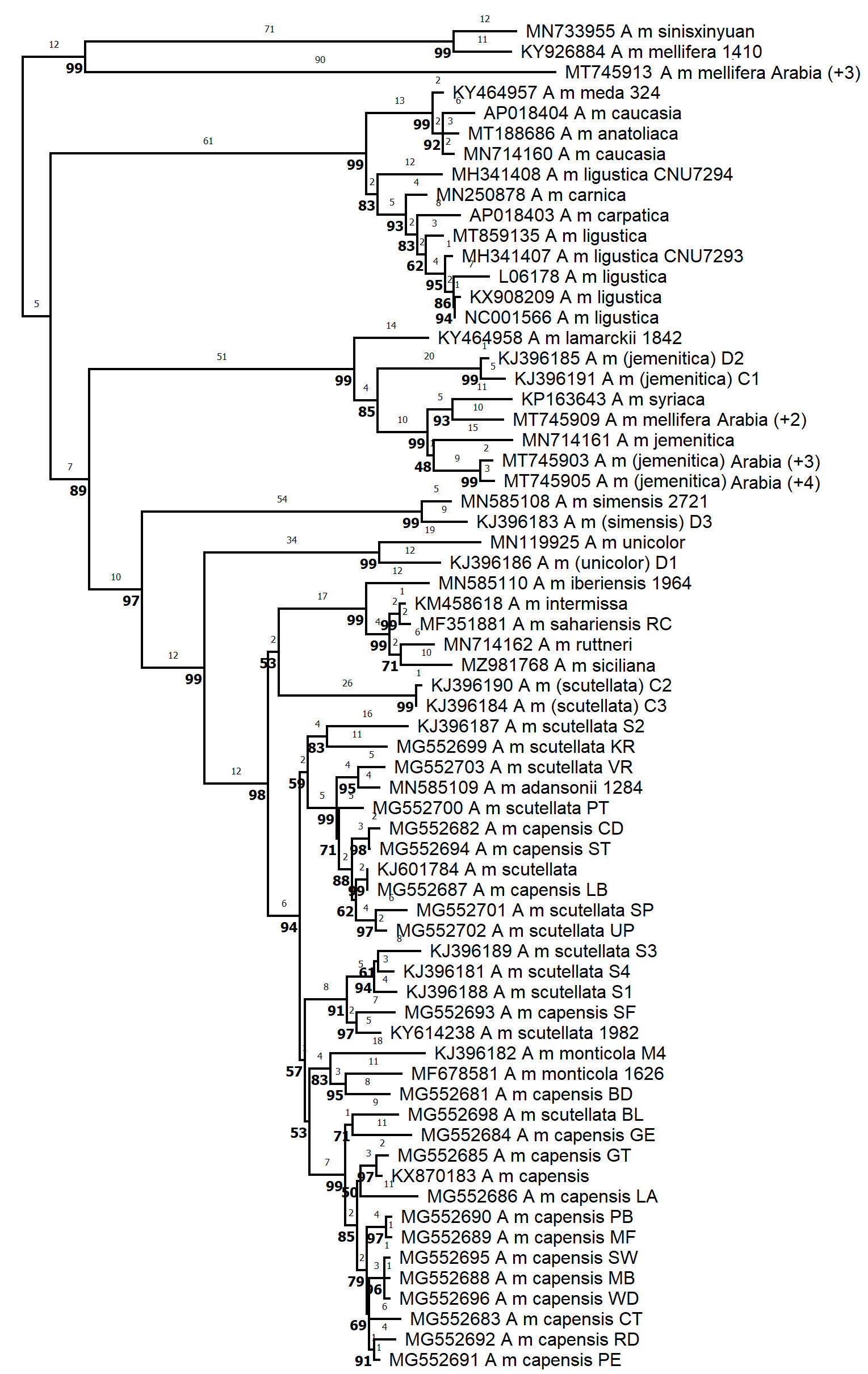

Supplement: Supplementary file 4 — Supplementary Information 4. [file 41598_2023_35937_MOESM4_ESM.jpg]

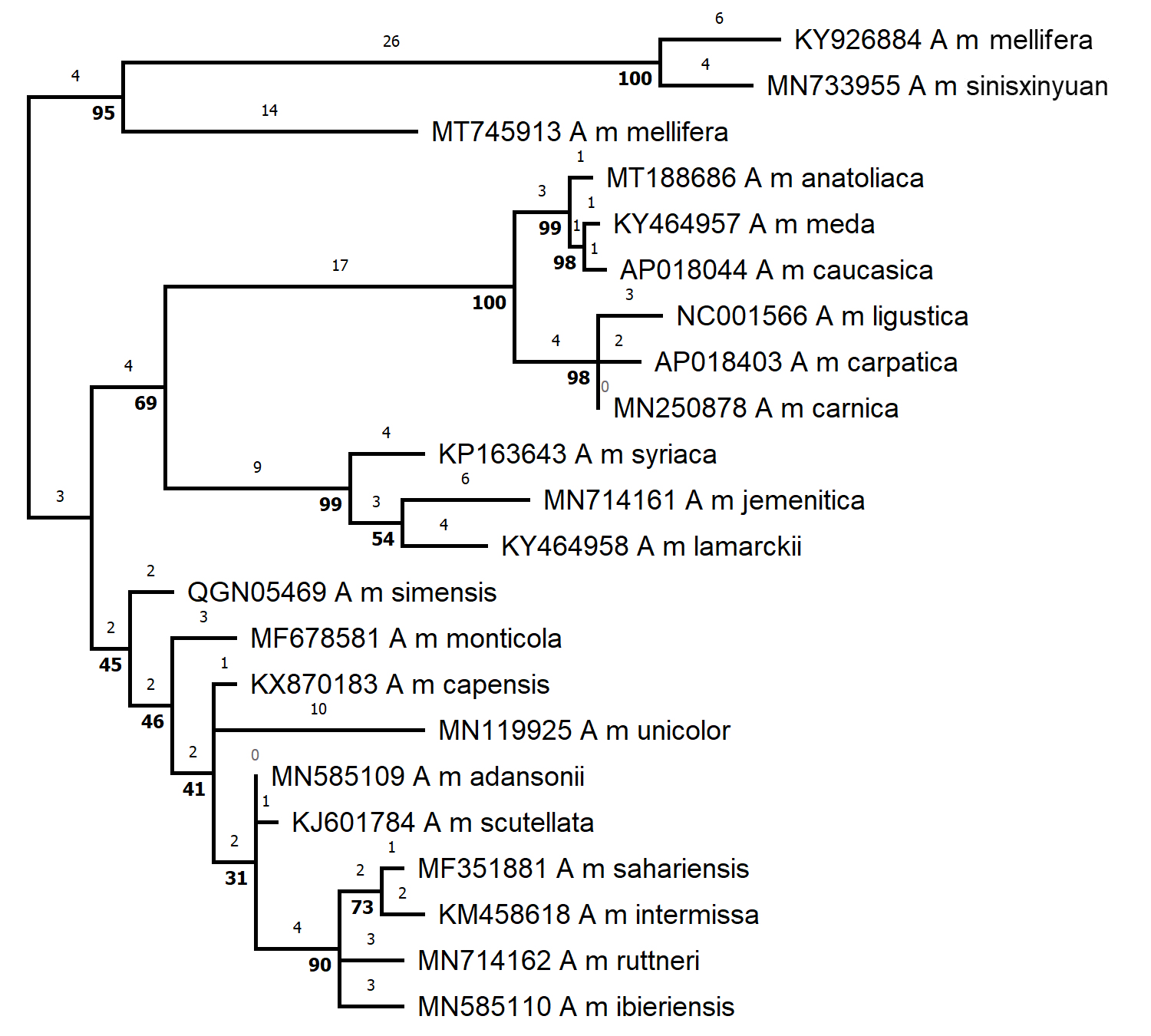

Supplement: Supplementary file 5 — Supplementary Information 5. [file 41598_2023_35937_MOESM5_ESM.jpg]

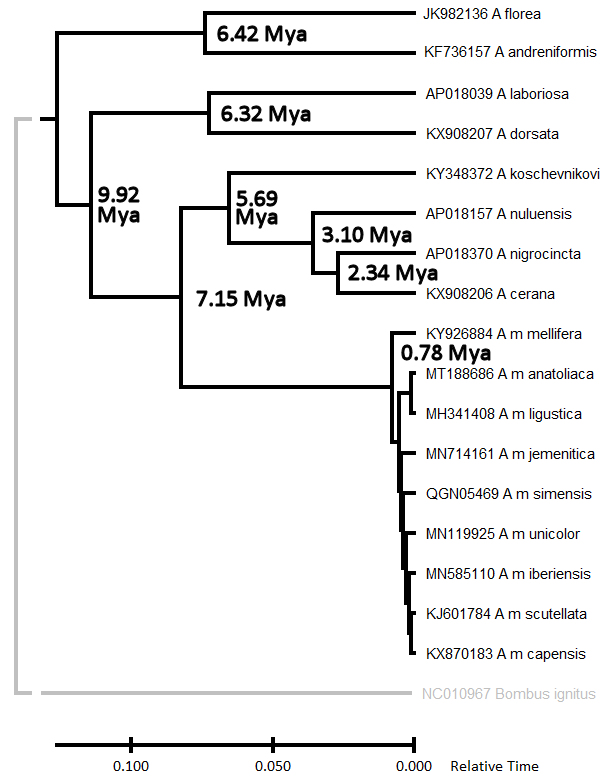

Supplement: Supplementary file 6 — Supplementary Information 6. [file 41598_2023_35937_MOESM6_ESM.jpg]

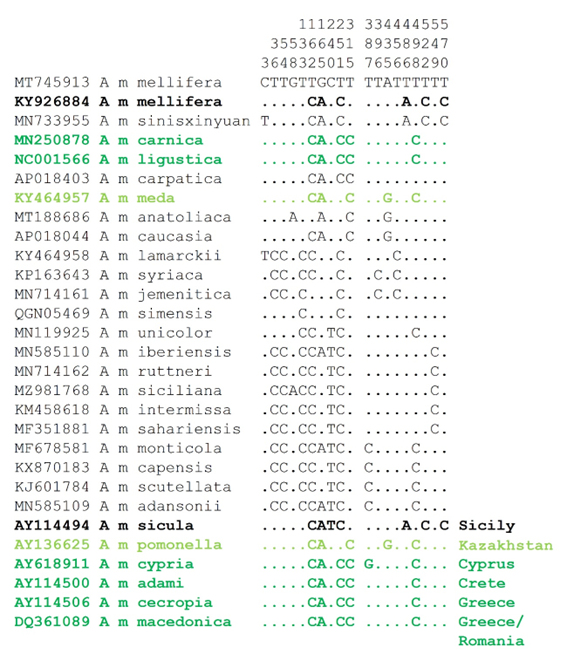

Supplement: Supplementary file 7 — Supplementary Information 7. [file 41598_2023_35937_MOESM7_ESM.jpg]

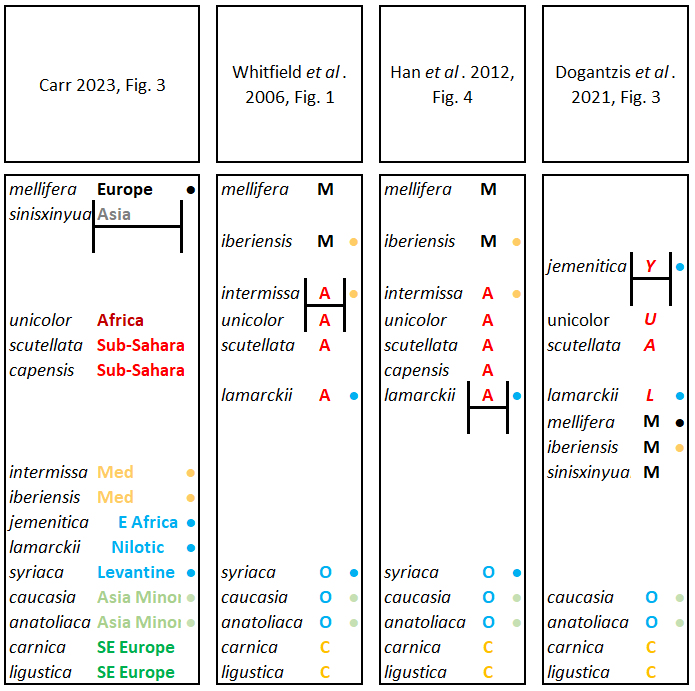

Supplement: Supplementary file 8 — Supplementary Information 8. [file 41598_2023_35937_MOESM8_ESM.jpg]
